# Parental acceptability of newborn screening expansion in the genomic era: A nationwide French survey informed by the Theoretical Framework of Acceptability (SeDeN-p3)

**DOI:** 10.1371/journal.pone.0343754

**Published:** 2026-06-15

**Authors:** Camille Level, Laurence Faivre, Margot Lemaitre, Dominique Salvi, Isabelle Marchetti-Waternaux, Elisabeth Cudry, Emmanuel Simon, Nicolas Bourgon, Alexandra Benachi, Nhut-Thanh Van, Camille Coppola, Christine Binquet, Christel Thauvin-Robinet, Frédéric Huet, Christine Peyron

**Affiliations:** 1 Université Bourgogne Europe, Dijon, France; 2 Fédération Hospitalo-Universitaire TRANSLAD, Centre Hospitalier Universitaire de Dijon Bourgogne, Dijon, France; 3 Centre de Génétique, Centre Hospitalier Universitaire de Dijon Bourgogne, Dijon, France; 4 Association Valentin des Porteurs d’Anomalies Chromosomiques, Eragny sur Oise, France; 5 Association Vaincre les Maladies Lysosomales, Massy, France; 6 Maternité, Centre Hospitalier Universitaire de Dijon Bourgogne, Dijon, France; 7 Service d’Obstétrique, Maternité Chirurgie, Médecine et Imagerie fœtales, Hôpital Necker-Enfants malades, Groupe Hospitalier Universitaire Paris Centre, Assistance Publique – Hôpitaux de Paris, Paris, France; 8 Service de Gynécologie-Obstétrique, Hôpital Antoine-Béclère, Assistance Publique – Hôpitaux de Paris, Clamart, France; 9 Groupe Hospitalier de la Haute-Saône, Vesoul, France; 10 Centre d’Investigation Clinique, module épidémiologie clinique, Institut National de la Santé Et de la Recherche Médicale, Unité 1432, Centre Hospitalier Universitaire de Dijon Bourgogne, Dijon, France; 11 Laboratoire de Génomique Médicale, Institut National de la Santé Et de la Recherche Médicale, Centre Translationnel Médical, Unité Mixte de Recherche 1231, équipe Génétique des Anomalies du Développement, Centre Hospitalier Universitaire de Dijon Bourgogne, Dijon, France; 12 Service de Pédiatrie, Centre Régional de Dépistage Néonatal, Centre Hospitalier Universitaire de Dijon Bourgogne, Dijon, France; 13 Laboratoire d’Économie de Dijon, Université Bourgogne Europe, Dijon, France; Athens Medical Group, Psychiko Clinic, GREECE

## Abstract

**Background:**

Newborn screening (NBS) has progressively expanded through technological innovations, from tandem mass spectrometry enabling expanded NBS (eNBS) to the prospect of genomic NBS (gNBS). While these developments promise earlier diagnosis and richer information, they also raise concerns regarding actionability, uncertainty, equity and psychosocial impact. As technological feasibility alone does not ensure public confidence, parental perspectives are central to evaluating future expansions. This study assessed parental views on NBS expansion in France, examining its determinants and whether genomics raises specific concerns.

**Methods:**

A nationwide cross-sectional survey (September 2022–February 2023) included 1,640 parents recruited postpartum in maternity wards and through an online quota panel. Acceptability of eNBS and gNBS was assessed alongside intermediate components from the Theoretical Framework of Acceptability (affective attitude, perceived effectiveness, ethicality), a technical trade-off scenario, and individual characteristics. Analyses combined descriptive statistics, multivariable regression, and thematic analysis of free-text comments.

**Results:**

Support was very high for eNBS (93%) and remained high for gNBS (89%), with genetics mainly shifting responses from complete to partial acceptability. Affective attitude and perceived effectiveness were the strongest predictors of both outcomes, while ethical concerns distinguished assured from conditional support. Most parents prioritised minimising uncertain results, whereas a smaller subgroup accepted greater ambiguity. Foreign-born and single parents reported lower levels of complete acceptability, while health-sector workers and parents with rare-disease experience were more supportive. No independent association with the age of the youngest child was observed.

**Conclusion:**

Parental acceptability of eNBS and gNBS is high but nuanced, shaped primarily by anticipated health benefits, emotional orientation and tolerance for uncertainty, with trust and social distance modulating support. As genomic expansion progresses, implementation will require proportionate, culturally adapted information and clear governance, and should be informed by real-world evidence from pilots such as PERIGENOMED.

## Introduction

### Newborn screening as an evolving public health programme

Newborn screening (NBS) is widely regarded as one of public health’s major achievements. Since the introduction of Guthrie’s phenylketonuria test and the formulation of the Wilson and Jungner principles in the late 1960s, high-income countries have progressively implemented universal programs to identify treatable disorders early in life. Over recent decades, the scope of NBS has widened with successive technological innovations [1]. Expanded NBS (eNBS), driven by tandem mass spectrometry and related methods, made it possible to screen simultaneously for multiple biochemical disorders and to progressively widen national panels. More recently, the introduction of genomic approaches in NBS (gNBS), encompassing targeted sequencing panels, exome and genome sequencing, has been presented as a further step change, enabling detection of hundreds of genetic conditions, including those without biochemical markers, and addressing some of the technical limits of standard screening in premature or ill newborns [2–4]. The advantages of such extensions include earlier diagnosis, where effective therapies are available and accessible, timely therapies, together with the potential contribution of population-level evidence to future research. Yet unresolved challenges (how to define actionability, manage uncertainty, limit false positives, ensure equity, and address psychosocial impacts) have been identified and debated for over a decade across successive waves of NBS expansion, and are now being revisited with added intensity in the genomic context [5–10]. Ongoing real-world pilots provide initial evidence on how these longstanding concerns translate into practice [11]. In this context, understanding how these developments are appraised becomes crucial, as these judgements ultimately determine what is regarded as acceptable within a public health programme. Recent work also underscores that the perspectives of stakeholders are essential to understanding under which conditions such technological shifts can be responsibly implemented [12,13].

### The relevance of test acceptability in NBS

Acceptability offers a relevant framework for analysing how individuals assess future forms of NBS, as it refers to the way they weigh anticipated benefits and burdens across cognitive, affective and normative dimensions [14]. This prospective perspective differs from acceptance, which relates to lived experience after rollout [15], and is particularly pertinent in NBS, where decisions are made upstream of any concrete exposure. Work applying the Theoretical Framework of Acceptability (TFA) has highlighted the central role of perceived appropriateness and shown that judgements depend on how users understand an intervention’s purpose, mechanisms and implications [16]. Within this landscape, parents hold a distinctive position: they are both the recipients of reassurance and the mediators of consent on behalf of their newborn. Studies of standard NBS illustrate that even in systems with near-universal uptake, participation is shaped by values, norms and perceptions of legitimacy, rather than by technical performance alone. Concerns related to pain, alternative medicine, religious beliefs or institutional trust illustrate how these orientations guide parental decision-making [17]. These observations echo broader insights from health services research, where acceptability is shown to vary across contexts according to perceived benefits, familiarity with the modality, confidence in handling its informational implications, and understanding of how the intervention works [18–20]. A large body of behavioural research also indicates that evaluative judgements can be sensitive to framing effects, where simple linguistic cues activate heuristics that shift perceptions of benefit and risks [21]. In this perspective, genetics may carry symbolic and informational weight, as genetic information is often associated with notions of heredity, identity and long-term implications, and may require higher levels of literacy or interpretive effort. Such representations can shape how parents anticipate potential benefits and burdens.

### International evidence on parental acceptability

In the case of NBS, international studies have explored parental views on eNBS (for example, Canada: [22]; Hong Kong [23]; Netherlands [24]; USA [25,26]) or gNBS ((Australia [27,28]; Canada [29,30]; Germany [31]; Slovenia [32]; UK [33]; United Arab Emirates [34]; USA [35,36]), but most have examined these modalities separately and without explicitly comparing how the nature of the test shapes parental evaluations. Synthesising this work, recent reviews describe parental acceptability as insufficiently theorised and often treated descriptively, with many studies relying on hypothetical scenarios or small samples and neglecting the influence of health-system structures and cultural contexts [37]. Yet contextual factors consistently appear to shape parental views [17]. The French setting, characterised by a strong public health tradition, near-universal participation in standard NBS and structured national governance, provides a particularly informative environment to examine how parents evaluate different modalities of NBS, and whether the informational and symbolic features associated with genomic testing activate specific concerns or reinforce evaluative patterns observed elsewhere [38–41].

### The SeDeN project and objectives of this article

This study is part of SeDeN (*Séquençage Dépistage Néonatal*), a multi-component research project examining the conditions under which eNBS and gNBS may be socially acceptable in France. The project comprises four complementary strands: SeDeN-P1, addressing national and international public policies; SeDeN-P2, exploring the views of perinatal and genetics professionals; SeDeN-P3, investigating parental perspectives in the general population and among families affected by rare diseases; and SeDeN-P4, focusing on policymakers and influential stakeholder groups. This article reports findings from SeDeN-P3, specifically the national survey of parents in general population. It pursues two main objectives: first, to identify the determinants of parental acceptability of NBS expansion by explicitly distinguishing the effect of test modality. Second, to characterise patterns of adherence and reservations to determine whether genomic techniques introduce specific concerns or reinforce general evaluative logics observed in eNBS. By comparing both modalities within the same respondents and situating the analysis upstream of a planned national pilot, this study aims to inform future discussions on communication strategies, consent frameworks, and policy options for forthcoming expansions of NBS.

## Materials and methods

### Study design

SeDeN-P3 was a nationwide cross-sectional survey conducted in France between September 2022 and February 2023 using a self-administered questionnaire, administered both online and on paper depending on the recruitment setting. The study followed a convergent mixed-methods design of the “validating quantitative data” type with a concurrent triangulation structure [42], in which quantitative data formed the core and qualitative inputs from open-text responses were used to corroborate and enrich interpretation.

This article focuses on parental acceptability of test modalities in NBS. Components addressing criteria for disease inclusion and parental expectations regarding information and consent are reported separately.

### Conceptual framework and outcomes

Acceptability was conceptualised as a multidimensional evaluative judgement combining anticipated benefits, perceived burdens, alignment with personal values, preferences for techniques and individual characteristics. We relied on the TFA proposed by Sekhon et al., which defines acceptability as a multifaceted construct reflecting the extent to which individuals receiving a health intervention consider it appropriate [14]. This framework was complemented by findings from the international literature on eNBS and gNBS available at the time of questionnaire design and refined through iterative discussions within a multidisciplinary research team (genetics, paediatrics, public health, midwifery, social sciences, and patient organisations).

**Fig 1:**
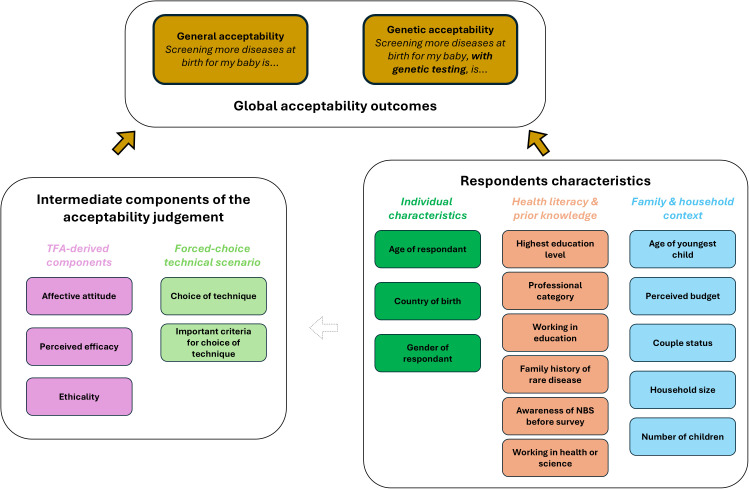
Conceptual framework of acceptability in our study. Fig 1 presents the structure of the key variables in our survey, organised into three interrelated blocks. Arrows indicate the hypothesised influence of these factors on overall and genetic acceptability ratings.

Fig 1 presents the conceptual framework guiding the study. It organises variables into blocks representing global acceptability outcomes, intermediate components of the acceptability judgement, and respondent characteristics.

Two global acceptability outcomes were assessed using parallel items adapted from Sekhon’s generic acceptability measure [43]. Parents rated on a 5-point Likert scale (from *Completely unacceptable* to *Completely acceptable*): (1) “Screening more diseases at birth for my baby is…”, then (2) the same statement specifying that the extension would rely on “a genetic test, through a DNA analysis”. This paired design aimed to isolate the effect of explicitly framing the extension as genetics. An optional free-text box followed the second item. Three intermediate TFA components relevant to a one-off preventive intervention were measured directly on 5-point Likert scales: affective attitude (emotional response to the intervention), perceived effectiveness (extent to which the intervention is expected to achieve its purpose), and ethicality (fit with personal values). A forced-choice technical scenario additionally asked parents to choose between three options trading off screening breadth and test precision, and to indicate the criterion guiding their choice; an optional free-text box allowed elaboration. Respondent characteristics covered individual attributes (age, sex, education, occupation), health literacy and prior knowledge (presence of a rare or genetic disease in the family, prior personal experience with genetic testing, prior information on NBS), and family and household context (marital status, number of children, perceived financial situation).

### Survey instrument

The questionnaire was developed iteratively by the multidisciplinary research team and refined through cognitive pretesting (structured interviews designed to verify respondents’ comprehension, recall and response strategies for each item) conducted in maternity wards and with panel participants prior to finalising the questionnaire. Revisions included simplifying medical terminology (e.g., replacing “genome sequencing” with “genetic testing”), shortening item stems to reduce cognitive load, and adjusting response formats for clarity. All core items were mandatory in the electronic version to minimise missing data. The final questionnaire (administered in French and translated into English for publication purposes) is available in S1 File.

### Participants and recruitment

Two complementary populations were recruited to reflect the range of contexts in which parents form judgements about eNBS and gNBS. Population 1 included parents of newborns ≤ 7 days, recruited exhaustively in four maternity wards between 09/09/2022 and 30/11/2022, approximating the real decision context at birth.

Recruitment occurred during predefined on-site inclusion days rather than continuously across the full period. Sites were selected to maximise diversity in geographic location, facility type and birth volume. Population 2 included parents of children aged 7 days-36 months, recruited via an online panel (*CPA Études*) between 04/01/2023 and 13/02/2023, using soft quotas on region, age and education approximating the parent population in metropolitan France.

In both populations, eligible participants were parents (biological or otherwise) residing in metropolitan France, not under judicial protection, and aged 18–50 years for mothers and 18–60 years for fathers (reflecting French reproductive patterns, available population benchmark categories for quotas, and preserving inclusion of older fathers). Each eligible parent could participate independently, including both partners within the same household. Exclusion criteria in Population 1 were neonatal death during recruitment and linguistic or cognitive barriers preventing autonomous completion of the questionnaire in French (routinely assessed by the attending healthcare team according to ability to read the information notice and respond without assistance).

This dual recruitment strategy reduced the selection bias typical of voluntary online surveys while capturing potential variation in perceptions as children age. Analyses were stratified by age of the youngest child rather than recruitment modality into four groups (≤7 days, 7 days–12 months, 12–24 months, 24–36 months). A minimum target of 385 participants was retained for each group (95% confidence level, 5% margin of error, 50% acceptability estimate). For Population 2, recruitment could continue beyond this threshold where needed to satisfy quota constraints.

Further details on site-level recruitment, inclusion periods and eligibility assessment are provided in S2 Table.

### Data analysis strategy

Data were collected via a smartphone-compatible electronic questionnaire. For Population 1, paper forms were also available; these were double-entered to minimise transcription errors. The anonymised dataset was stored on the CHU Dijon Bourgogne server and was accessible only to authorised study personnel. For optional sociodemographic variables, analyses were conducted using available responses without imputation of missing data.

Given the absence of full psychometric validation of the TFA-based items and ongoing methodological debate regarding the treatment of 5-point Likert scales [44–47], all items were analysed as ordinal categorical variables. Univariate analyses described response distributions for the two global acceptability outcomes and for all intermediate components with percentages and absolute frequencies. Non-paired associations between global acceptability outcomes, intermediate components and respondent characteristics were tested using Pearson’s χ² or the Fisher-Freeman-Halton exact test when Cochran’s rule was violated. Effect sizes were quantified using Cramer’s V (V < 0.10 very weak; 0.10–0.19 weak; 0.20–0.29 moderate; ≥ 0.30 strong). Paired differences between eNBS and gNBS ratings were examined using the Wilcoxon signed-rank test (α = 0.05), with respondents classified as showing an unchanged, increased or decreased rating.

Predictors of acceptability were examined through separate logistic regression models for eNBS and gNBS. The primary outcome was a binary variable contrasting positive acceptability (*Somewhat acceptable* or *Completely acceptable*) with non-positive responses (*Somewhat unacceptable* or *Completely unacceptable*). Because most decreases between eNBS and gNBS occurred within the positive range, complementary models distinguishing *Completely acceptable* from *Somewhat acceptable* were also estimated (see Results and S3 Tables). Neutral responses were excluded from modelling due to their heterogeneous interpretation (genuine indecision, ambivalence or uncertainty).

Candidate predictors included TFA-derived components, the criterion guiding the technical trade-off decision, and individual characteristics associated with the outcome at p ≤ 0.10, consistent with exploratory aim. Models were screened for multicollinearity (variance inflation factor (VIF)<5 for continuous predictors; Cramer’s V < 0.30 for categorical associations), and ranked using Akaike Information Criterion (AIC). Results are reported as adjusted odds ratios (OR) with 95% confidence intervals (CI). Detailed specifications, diagnostic statistics and complementary model comparisons are provided in S3 Table.

#### Qualitative analysis and mixed-methods integration.

Qualitative material from two open-text boxes was analysed thematically by two independent researchers with backgrounds in economics and sociology. Following Creswell’s guidance for convergent mixed methods designs, qualitative and quantitative strands were analysed separately and integrated during interpretation. Themes were compared across levels of eNBS and gNBS acceptability and across technical preferences to identify convergences and divergences. Illustrative quotations are presented in the Results when they clarify or contextualise quantitative patterns. All quoted extracts were reviewed to ensure that no personal identifier, demographic specificity or any information that could reasonably identify a respondent was included; this requirement was anticipated in the information notice, which explicitly asked participants not to enter any personal or identifying information in free-text boxes beyond what was explicitly requested.

Open-text comments were mapped onto a two-dimensional matrix defined by respondents’ paired eNBS and gNBS acceptability ratings. Each comment was represented as a bubble positioned according to the combination of ratings, with bubble size reflecting the number of respondents sharing the same rating configuration. This visual mapping was used as an exploratory tool to support the identification of qualitative profiles.

#### Software.

Quantitative analyses were conducted using Microsoft Excel (version 2506) and R *via* RStudio (version 4.4.2). Qualitative data management and thematic analysis were performed with NVivo 12 Plus. Data cleaning support was provided by ADN Soft via the HARMONIE platform.

#### Ethics approval and consent to participate.

The protocol, data collection tools, and information sheets for families were approved by the Ethics Committee for Research of the University of Burgundy-Franche-Comté on September 8, 2022 (reference CERUBFC-2022-04-27-015). The study was conducted under the French CNIL MR-004 reference methodology, which governs non-interventional health research involving routinely collected or questionnaire data; this framework does not require written consent but relies on documented non-opposition. Participation was voluntary. All participants received written information describing the study objectives, procedures, data collected, and their rights. Completion of the questionnaire, following receipt of the information notice, constituted non-opposition to the processing of responses for research and publication purposes. For Population 1, information was provided on-site by trained research staff during the maternity stay, using a written information and non-opposition notice. For Population 2, the same information was provided on the first page of the online questionnaire. Data were collected anonymously and analysed in accordance with applicable data protection regulations (Regulation (EU) 2016/679 and French Law 78−17 of 6 January 1978 as amended).

## Results

### Recruitment and respondent characteristics

**Fig 2:**
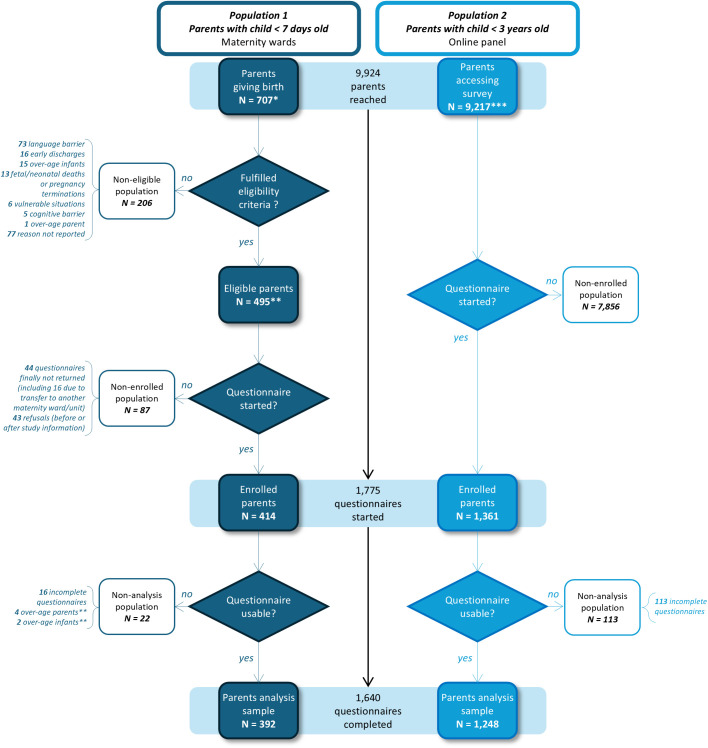
Participant flow for the two recruitment populations. * Based on national estimates from the 2021 DREES survey on childcare arrangements (https://drees.solidarites-sante.gouv.fr/sources-outils-et-enquetes/lenquete-modes-de-garde-et-daccueil-des-jeunes-enfants), 89.7% of parents of children under age 3 in metropolitan France live in a two-parent household. Applying this proportion to the 373 deliveries recorded during the recruitment period in Population 1, we estimate that approximately 707 parents could theoretically have been reached to complete the questionnaire. This estimate accounts for both two-parent households (two potential respondents per delivery) and single-parent households (one respondent) and was used to define the upper bound of potential respondents for coverage calculations. ** Of the 22 non-analysed cases, 6 were found post hoc to be ineligible (4 outside age range, 2 with a child older than 7 days) and were therefore excluded from the denominator of eligible participants to ensure consistency with inclusion criteria. *** In Population 2, 9,217 individuals accessed the survey page. The estimated parent population is not directly known. Among them, 1,361 answered the first substantive question, representing the starting point for inclusion. The gap with initial clicks (n = 7,854) reflects early exits, quota ineligibility, or disengagement before consent or first question.

A total of 1,640 parents were included in the analytical sample (Population 1: n = 392; Population 2: n = 1,248), with an overall completion rate of 92.4%. Recruitment flow for both populations is shown in Fig 2. Respondent characteristics are presented in Table 1.

**Table 1:** Respondents’ characteristics, by recruitment site and population group, with reference values.

	Population 1* N = 392	Population 2* N = 1,248	Total* N = 1,640	Reference value**
**Individual characteristics**				
**Gender of respondent**				
Female	227 (63%)	903 (72%)	1,130 (70%)	*50.5%* ^*1*^
Male	134 (37%)	345 (28%)	479 (30%)	*49.5%*
**Age of respondent**	mean: 32,47	mean: 34,05	mean: 33,67	
< 25 years	30 (7.7%)	60 (4.8%)	90 (5.5%)	*20.60%* ^*2*^
25-35 years	226 (58%)	580 (46%)	806 (49%)	*28.80%*
> 35 years	132 (34%)	608 (49%)	740 (45%)	*50.60%*
**Country of birth**				
France	268 (85%)	1,106 (93%)	1,374 (91%)	*87.2%* ^*3*^
Foreign country	48 (15%)	85 (7.1%)	133 (8.8%)	*12.8%*
*Other* *European* *country*	*13 (4.1%)*	*24 (2.0%)*	*37 (2.5%)*	
*African country*	*30 (9.5%)*	*46 (3.9%)*	*76 (5.0%)*	
*Asian country*	*2 (0.6%)*	*9 (0.8%)*	*11 (0.7%)*	
*American country*	*3 (0.9%)*	*6 (0.5%)*	*9 (0.6%)*	
**Health literacy & prior knowledge**				
**Working in science**	60 (17%)	177 (15%)	237 (16%)	*6.1%* ^*4*^
**Working in health**	57 (16%)	156 (13%)	213 (14%)	*6.9%* ^*4*^
**Working in teaching field**	13 (3.6%)	76 (6.6%)	89 (5.9%)	*7.3%* ^*4*^
Professional category ***				
Not working	na	217 (17%)	217 (17%)	*6.9%* ^*5*^
Lower occupational class	na	542 (43%)	542 (43%)	*44.9%*
Upper occupational class	na	489 (39%)	489 (39%)	*48.2%*
**Highest education level**				
Less than high school	78 (20%)	178 (14%)	256 (16%)	*30%* ^*4*^
High school to bachelor’s	183 (47%)	762 (61%)	945 (58%)	*49.2%*
Master’s or higher	125 (32%)	308 (25%)	433 (26%)	*20.9%*
**Awareness of NBS before survey**				
Not knowing NBS	208 (54%)	586 (47%)	794 (49%)	
Knowing NBS	178 (46%)	662 (53%)	840 (51%)	
**Family history of rare disease**				
Yes, family affected by a rare disease	45 (13%)	170 (15%)	215 (14%)	
No/Unsure	298 (87%)	999 (85%)	1,297 (86%)	
**Family & household situation**				
**Couple status**				
In couple	367 (97%)	1,129 (91%)	1,496 (93%)	*89.7%* ^*2*^
Single parent	11 (2.9%)	110 (8.9%)	121 (7.5%)	*10.3%*
**Household size**				
1-2 people	33 (8.9%)	132 (11%)	165 (10%)	
3 or more people	338 (91%)	1,113 (89%)	1,451 (90%)	
Number of children ****				
1 child	163 (47%)	506 (41%)	669 (43%)	*32.8%* ^*2*^
2 children	129 (37%)	432 (35%)	561 (36%)	*41.2%*
3 or more children	53 (15%)	288 (23%)	341 (22%)	*26.1%*
**Age of the youngest child**				
< 1 week	392 (100%)	12 (1.0%)	404 (25%)	*35.7%* ^*2*^
1 week – < 1 year	na	414 (33%)	414 (25%)	
1 – < 2 years	na	423 (34%)	423 (26%)	*31.3%*
2 - 3 years	na	399 (32%)	399 (24%)	*33.0%*
**Perceived budget**				
Struggling	4 (1.1%)	148 (12%)	152 (9.7%)	
Needs monitoring	69 (20%)	367 (30%)	436 (28%)	
Balanced	178 (50%)	541 (45%)	719 (46%)	
Comfortable	102 (29%)	159 (13%)	261 (17%)	

* Totals are counts and percentages of valid cases (excluding missing data); ** To assess sample representativeness, we drew on five data sources in priority order, using statistics for parents of children < 3 years when available, otherwise the general 18–50 year-old population: 1. INSEE, Demographic Report 2022 (2021 data): individuals 18–50 years (N = 2,70.10^7^); 2. DREES, Childcare Survey 2021: parents of children < 3 years (N = 9,000); 3. INSEE, Population Estimates 2021: total population census; 4. INSEE, Employment 2021: individuals 25–45 years and 25–49 years (N = 2,26.10^6^); 5. INSEE, Couples & Families 2021: parents of children < 3 years (N = 2,08.10^6^); *** This variable was used to enforce mandatory quotas for Population 2 and was not included in the questionnaire for maternity-ward respondents (Population 1) For professional category, defined as Upper occupational class (n = 489; 39%): managers and higher intellectual professions (n = 254), intermediate professions (n = 180), craftspeople and small business owners (n = 47), and farmers (n = 8); Lower occupational class (n = 542; 43%): clerical and service workers (n = 474) and manual workers (n = 68); Not working (n = 217; 17%): respondents not currently in employment (students, homemakers, unemployed, retired); **** Newborn included if questionnaire completed in maternity ward; na: not applicable.

### Global acceptability of NBS expansion and effect of genomic framing

93.2% (n = 1,528) of respondents rated the idea of *Screening more diseases at birth for their child* as *Somewhat acceptable* or *Completely acceptable*. When the genetic dimension was explicitly introduced (by specifying *using a genetic test, DNA analysis*), this general level slightly declined to 89.3% (n = 1,463). The difference was statistically significant (Wilcoxon paired test, p < 0.0001) but reflected a shift in intensity rather than a reversal of direction.

**Fig 3:**
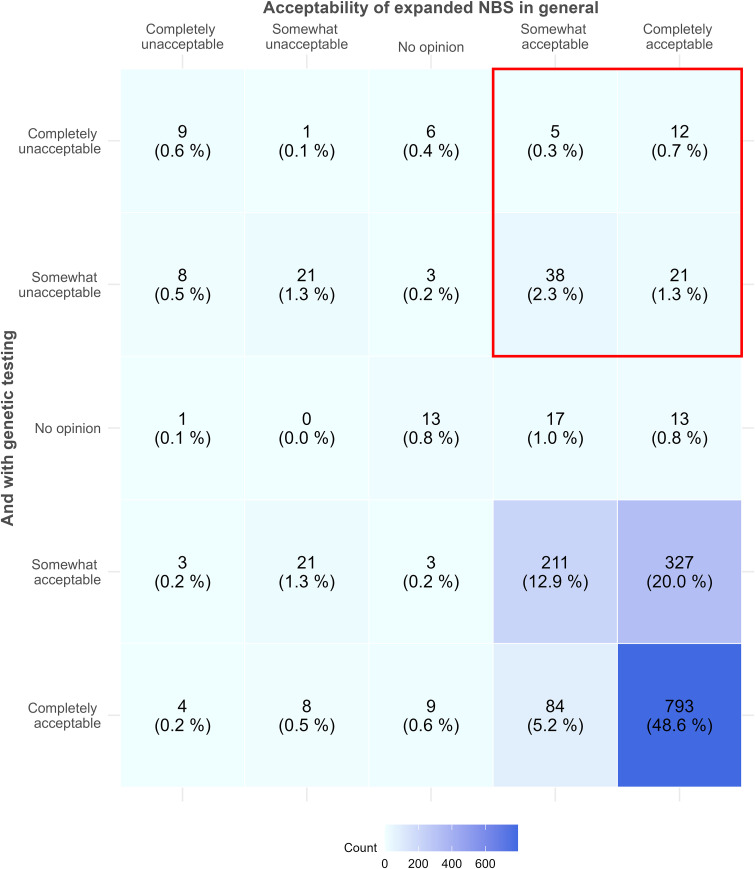
Cross-distribution of responses to general and genomic expanded newborn screening acceptability. Heatmap of paired responses (N = 1,631) showing high overall acceptability for both scenarios. Most participants maintained a positive opinion. The red box highlights downward transitions from a previously supportive stance (n = 76; 4.6%).

Among the 1,631 participants who answered both items, 64.2% (n = 1,047) gave identical ratings, including 48.6% (n = 793) who consistently chose *Completely acceptable* (Fig 3). Most changes observed in the remaining third of respondents were modest, with 27.1% (n = 443) moving downwards by one category (most often from *Completely acceptable* to *Somewhat acceptable*, n = 327; 20.0%). Only 4.6% (n = 76) moved from a positive to a negative category, while 8.6% (n = 141) shifted upwards.

Acceptability remained high across groups defined by the age of the youngest child, including the immediate postpartum period, with positive ratings above 90% for eNBS and above 85% for gNBS. Differences were statistically significant for eNBS (p < 0.001) and gNBS (p = 0.014), with small effect sizes (resp. Cramer’s V = 0.09 and 0.07), and mainly reflected modest shifts between *Completely acceptable* and *Somewhat acceptable* (Table 2).

**Table 2:** Global acceptability of eNBS and gNBS by age of the youngest child.

Global acceptability of eNBS by age of the youngest child					
Age of youngest child	Completely unacceptable	Somewhat unacceptable	No opinion	Somewhat acceptable	Completely acceptable
< 1 week	1.7% (7)	1.0% (4)	2.2% (9)	15.7% (63)	79.4% (319)
1 week – < 1 year	2.9% (12)	3.6% (15)	1.2% (5)	22.9% (95)	69.3% (287)
1 – < 2 years	1.2% (5)	4.5% (19)	1.7% (7)	24.8% (105)	67.8% (287)
2 - 3 years	0.3% (1)	3.3% (13)	3.3% (13)	23.3% (93)	69.9% (279)
**Global acceptability of gNBS by age of the youngest child**					
**Age of youngest child**	**Completely** **unacceptable**	**Somewhat** **unacceptable**	**No opinion**	**Somewhat** **acceptable**	**Completely** **acceptable**
< 1 week	3.0% (12)	3.5% (14)	5.0% (20)	34.0% (135)	54.4% (216)
1 week – < 1 year	1.7% (7)	6.5% (27)	2.7% (11)	37.2% (154)	51.9% (215)
1 – < 2 years	2.4% (10)	7.1% (30)	0.9% (4)	32.6% (138)	57.0% (241)
2–3 years	1.0% (4)	5.3% (21)	2.5% (10)	34.6% (138)	56.6% (226)

Percentages (absolute numbers in parentheses) indicate parental ratings across five response categories, stratified by age of the youngest child. Differences across age groups were statistically significant for eNBS (χ² test, p < 0.001; Cramer’s V = 0.09) and gNBS (χ² test, p = 0.014; Cramer’s V = 0.07), indicating small effect sizes and limited practical variation.

### Intermediate components of the acceptability judgement

For affective attitude, 88.4% (n = 1,441/1,640) agreed or strongly agreed with the statement *I would like more diseases to be screened at birth for my baby*. For perceived effectiveness, 92.4% of respondents (n = 1,507) agreed or strongly agreed that *Screening more diseases at birth for [their] baby can improve [its] health*. Regarding ethicality, 62.2% (n = 1,014) disagreed or strongly disagreed that *Screening more diseases at birth for their baby raises moral or ethical concerns*, whereas 31.4% (n = 515) agreed or strongly agreed, indicating that nearly one third of parents perceived ethical questioning associated with eNBS.

When asked to choose between three hypothetical strategies 43.9% (n = 713) of parents selected the most restrictive but most reliable option (Technique C) and 55.0% (n = 892) reported that minimising uncertainty was their main criterion (Table 3).

**Table 3:** Distribution of parental screening strategy choices by decision criterion.

Important criteria for choice of technique	Overall N = 1,628^*1*^	Choice of technique		
		Technique A N = 320^*1*^	Technique B N = 595^*1*^	Technique C N = 713^*1*^
Number of diseases	255 (16%)	167 (53%)	54 (9.2%)	34 (4.8%)
Uncertainty risk	893 (55%)	53 (17%)	272 (46%)	568 (80%)
Both	472 (29%)	98 (31%)	263 (45%)	111 (16%)

^1^n (%).

The table shows the distribution of parental choices between the three hypothetical screening strategies (Techniques A, B, and C) according to the main criterion reported to guide their decision. Percentages are calculated by row (within each technique). “Uncertainty risk” refers to minimising ambiguous results, “Number of diseases” to maximising screening breadth, and “Both” to respondents indicating both criteria.

Parents prioritising uncertainty minimisation emphasised the risks of parental anxiety, stress, and potential psychological harm associated with uncertain or unreliable results, with typical comments such as: “*The results must be very reliable, otherwise they are too anxiety-inducing*” and “*False results can have a very serious impact on the child’s health but also on the parents’ mental health.*”. Respondents prioritizing the number of diseases screened tended to justify their choices by emphasizing the importance of maximizing early detection and clinical benefit and often acknowledging the need for confirmatory testing in case of positive results. Finally, those who selected both criteria typically advocated for a balance between broad screening and test reliability, frequently recommending combined or sequential testing approaches to ensure both comprehensive and reliable results. Anonymized coded verbatims are provided in S4 Table.

### Multivariable determinants of acceptability

**Fig 4:**
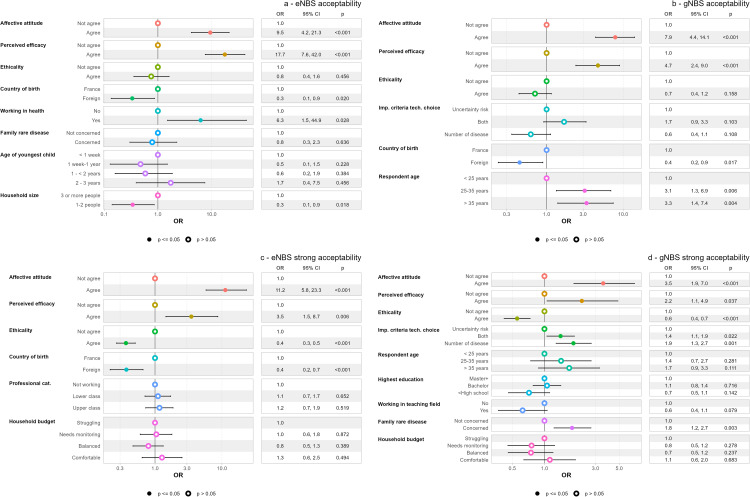
Multivariable regression forest plots for eNBS and gNBS acceptability. For each model, odds ratios (OR) and their 95% confidence intervals (CI) are shown for each explanatory variable (adjusted for all covariates included in the model). Filled circles indicate statistically significant associations (p ≤ 0.05); open circles represent non-significant results. The table to the right of each plot displays the exact OR, 95% CI, and p-value for each modality. Reference categories (OR=1) are shown at the top of each variable block. Models shown: 4a: General acceptability (*Completely acceptable/Somewhat acceptable* vs. *Completely unacceptable*/*Somewhat unacceptable*); 4b: Genomic acceptability (*Completely acceptable/Somewhat acceptable* vs. *Completely unacceptable*/*Somewhat unacceptable*); 4c: General strong acceptability (*Completely acceptable* vs. *Somewhat acceptable*); 4d: Genomic strong acceptability (*Completely acceptable* vs. *Somewhat acceptable*).

Logistic regression models distinguished two outcome contrasts: positive acceptability (*Somewhat/Completely acceptable* vs *Somewhat/Completely unacceptable*) and strong acceptability (*Completely acceptable* vs *Somewhat acceptable*), for each of the two scenarios. Forest plots of all four models are shown in Fig 4.

For positive acceptability of eNBS (Fig 4a), the two strongest predictors were perceived effectiveness (OR 17.7, 95% CI 7.6–42.0, p < 0.001) and affective attitude (OR 9.5, 95% CI 4.2–21.3, p < 0.001). Among respondent characteristics, foreign-born parents (OR 0.3, 95% CI 0.1–0.9, p = 0.020) and single parents (OR 0.3, 95% CI 0.1–0.9, p = 0.018) expressed positive support less often, while employment in the health sector was associated with stronger support (OR 6.3, 95% CI 1.5–44.9, p = 0.028).

For positive acceptability of gNBS (Fig 4b), affective attitude (OR 7.9, 95% CI 4.4–14.1, p < 0.001) and perceived effectiveness (OR 4.7, 95% CI 2.4–9.0, p < 0.001) remained the principal predictors, although the magnitude of perceived effectiveness was substantially attenuated. Foreign-born respondents again showed lower support (OR 0.4, 95% CI 0.2–0.9, p = 0.017). Parents aged 25–35 (OR 3.1, 95% CI 1.3–6.9, p = 0.006) and over 35 (OR 3.3, 95% CI 1.4–7.4, p = 0.004) were more likely to express support than parents aged 18–24. The criterion guiding the technical trade-off was not independently associated with positive acceptability in either model.

Models for strong acceptability provided additional refinements. In both eNBS and gNBS models, higher affective attitude (eNBS: OR 11.2, 95% CI 5.8–23.3, p < 0.001; gNBS: OR 3.5, 95% CI 1.9–7.0, p < 0.001) and perceived effectiveness (eNBS: OR 3.5, 95% CI 1.5–8.7, p = 0.006; gNBS: OR 2.2, 95% CI 1.1–4.9, p = 0.037) remained positive predictors of complete support. Ethicality then differentiated forms of adherence for eNBS (OR 0.4, 95% CI 0.3–0.5, p < 0.001) and showed a similar effect for gNBS (OR 0.6, 95% CI 0.4–0.7, p < 0.001). Foreign-born respondents were less likely to express strong support for eNBS (OR 0.4, 95% CI 0.2–0.7, p < 0.001). A family history of rare disease was associated with more frequent strong support for gNBS (OR 1.8, 95% CI 1.2–2.7, p = 0.003). Parents prioritising the number of diseases screened over uncertainty minimisation were also more likely to find gNBS completely acceptable (OR 1.9, 95% CI 1.3–2.7, p = 0.001; OR 1.4, 95% CI 1.1–1.9, p = 0.022 for the mixed criterion).

Age of the youngest child, including the immediate postpartum period, was not independently associated with acceptability in any of the four adjusted models.

### Qualitative material and mapping of parental profiles

**Fig 5:**
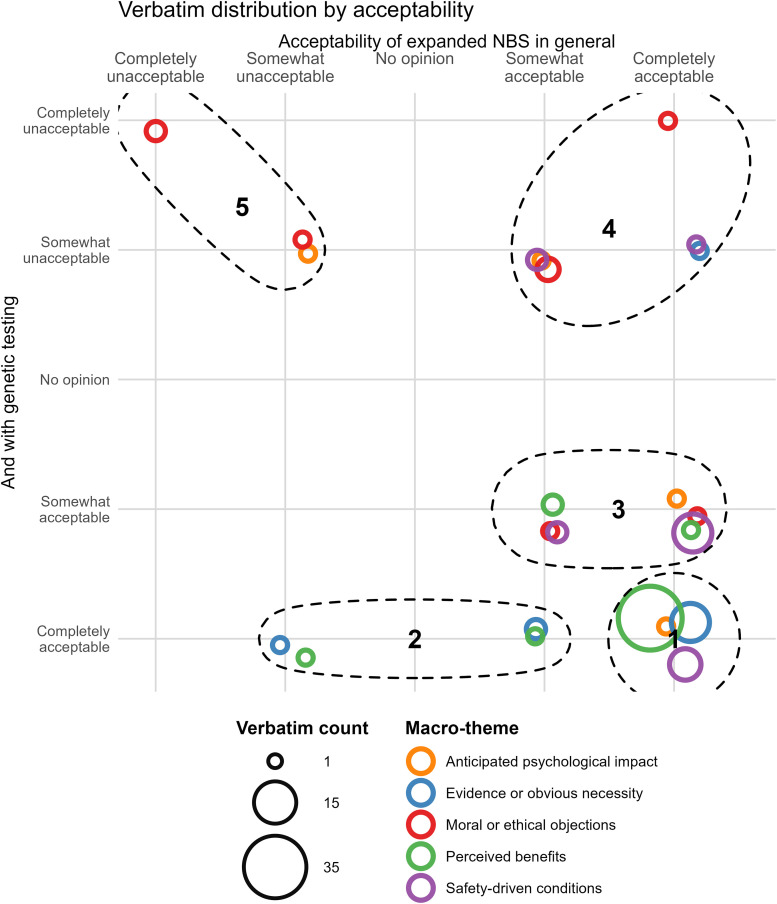
Mapping of parental comments by shift in acceptability between general and genomic NBS. Bubble plot mapping parental verbatim comments (n = 81) onto paired acceptability ratings of expanded (x-axis) and genomic newborn screening (y-axis). Each bubble represents one theme comment, positioned according to the respondent’s global acceptability scores. Bubble size is proportional to the number of respondents sharing the same position, while bubble colour indicates the main thematic category of the associated comment: (1) information and consent (orange), (2) perceived health benefits (blue), (3) concerns about uncertainty or psychosocial impact (red), (4) ethical or value-based considerations (green), and (5) broader views on science, society, and governance (purple). Dotted ellipses delineate the five shift zones (1-5). The grid of the plot defines distinct zones of movement: along the diagonal (stable acceptability between expanded and genomic versions), below it (decrease in acceptability when genetics is introduced), and above it (increase in acceptability).

Thematic analysis of the 81 unique free-text comments returned (4.9% of respondents; cross-tabulation of these respondents’ eNBS and gNBS ratings provided in S5b Table), mapped onto a two-dimensional matrix combining eNBS and gNBS acceptability ratings, revealed five recurrent profiles (Fig 5). A first profile gathered parents strongly favourable to both scenarios, whose judgements centred on anticipated benefits, the value of early diagnosis, and the usefulness of anticipatory information. A second profile expressed reinforced support when genetics was explicitly named, the prospect of genomic analysis amplifying an already favourable disposition. A third profile maintained positive evaluations under explicit conditions: non-invasiveness of the test, confidentiality of data, clear legal framing, and proportionate use. A fourth profile included parents favourable to the general extension who became more reserved when genetics was named, expressing concerns about privacy, eugenic risks, or insufficient current safeguards. The fifth profile gathered positions of unacceptability across both scenarios, grounded in ethical or existential objections. Detailed thematic coding and illustrative quotations are provided in S4 Table.

## Discussion

### Main findings and contribution

This nationwide survey of 1,640 French parents documents four findings. First, parental support remained high under explicit genomic framing; the observed change concerned the intensity of support, not its direction. Second, the structure of the acceptability judgement was largely preserved across modalities, with affective attitude and perceived effectiveness as the main predictors, but the weight of perceived effectiveness was substantially attenuated under genomic framing. Third, ethicality differentiated forms of adherence rather than generating opposition, predicting strong rather than positive acceptability, with a substantial minority expressing principled reservations despite an overall favourable stance. Fourth, sociodemographic and experiential factors modulated support: parents born abroad were less supportive, while parents with a family history of rare disease were more likely to find gNBS completely acceptable, an effect specific to the genomic framing. The age of the youngest child, including the immediate postpartum period, was not independently associated with acceptability.

These findings extend an international literature that has consolidated rapidly [13,37,48,49]. Whereas most prior studies addressed eNBS or gNBS separately, the present work uses a paired within-respondent design to isolate the effect of explicitly framing extension as genomic, and applies the TFA as an explicit conceptual framework.

### Why parents support expansion: anticipated gains, reassurance, and actionability

The high level of support documented here aligns with international evidence (S6 Table).

The few studies directly comparing conventional and genomic scenarios also show a recurrent attenuation rather than reversal. In Australia, support fell from 99% for standard NBS to 77% for gNBS [50]; in Canada, from 94.4% to 79.6% [51]; and in the United States, the BabySeq Project reported 78.7% strong agreement with universal screening in the control arm versus 71.0% in the gNBS arm [52]. Our paired design extends this pattern by documenting, for the first time at this scale in France, an internal shift toward more qualified rather than negative endorsement.

Anticipated health benefits, earlier diagnosis, improved prognosis, and the reassurance of knowing emerged as the main justifications, in line with prior work [22,27,31,34,35,38]. Information was valued for its anticipatory function as much as for its clinical utility.

### Uncertainty, reliability and ethical concerns

The strong preference for the most reliable option, the prioritisation of uncertainty minimisation, and the recurrent association in free text between uncertain results and parental anxiety document an aversion to uncertainty operating as a distinct evaluative logic.

Although such concerns were not dominant, some parents anticipated anxiety, loss of carefree parenting or altered parent–child relationships if uncertain results were returned. This pattern is consistent with earlier eNBS and gNBS studies reporting reservations about late-onset conditions, lower test accuracy, limited treatment options, ambiguous findings, and false positives [22,27,31,35,53]. For some families, informational gains may therefore be outweighed by anticipated psychosocial costs. A smaller subgroup, by contrast, appeared more tolerant of uncertainty and was more likely to judge gNBS completely acceptable, indicating heterogeneity in how parents weigh uncertainty against potential benefits.

Non-invasiveness also formed part of this calculus. Several parents explicitly mentioned discomfort linked to heel-prick procedures, noting that pain or mishandling could make screening feel disproportionate to its purpose. Similar reactions have been reported elsewhere [54]. Many parents nonetheless acknowledged that the health benefits of eNBS outweighed procedural inconvenience, a balance also observed elsewhere [4,55].

Within the TFA, these themes map onto burden and intervention coherence: acceptability depends not only on perceived benefit but also on the procedural and informational effort involved.

Ethical concerns formed a related but distinct dimension. Free-text comments under genomic framing revealed an enriched repertoire (autonomy, right not to know, data governance, discrimination, eugenic concerns), echoing the gNBS literature [27,34,38]. They have also been reported in eNBS, particularly around blood sample handling, pain and data use [17,23]. The fact that ethicality predicted strong rather than positive acceptability suggests that ethical concerns qualify endorsement rather than displace it; they identify a subgroup whose reservations are explicit and principled, particularly under genomic framing.

### Trust, experience and social position

Lower acceptability among foreign-born and single parents contrasted with greater support among health-sector workers and parents with experience of rare disease. The foreign-born effect was robust across three of the four regression models and is consistent with work on the importance of adapting information across linguistic and socioeconomic contexts [4,36]. The association between family history of rare disease and strong support specifically for gNBS is informative: prior personal exposure to diagnostic uncertainty appears to make the informational yield of gNBS particularly salient, whereas it does not modify support for the conventional extension. These patterns echo prior findings on familiarity, health literacy, and exposure to genetic conditions [25,32,56]. Parental judgement therefore appears socially situated.

### A behavioural economics reading: how parental judgement is structured across modalities

Read jointly, these results suggest that parental acceptability is structured by deliberative weighting of expected benefits and harms alongside attitudinal and informational factors that shape how such weighting is performed. The dominant weight of affective attitude is consistent with the role of attitudes in shaping intentions [25]: NBS as an established public health programme appears to provide a positive evaluative anchor for considering extensions. When expansion is explicitly framed as genetic, cues linked to uncertainty and informational complexity become more salient, and the intuitive shortcut linking expansion with clear benefit becomes less readily available, in line with framing processes [21]. Genetics thus introduces additional complexity rather than replacing prior orientations toward NBS. gNBS is evaluated through the acceptability already attached to conventional NBS, and support depends on the technology, on the legitimacy of the screening framework into which it would be integrated, and on the accessibility of the information through which parents are invited to consider it.

### Implications for implementation and governance

#### Communication and information.

Communication around NBS expansion should anchor on what structures parental judgement: anticipated child benefit and the management of uncertainty, rather than the technical sophistication of the test. Information on the categories of result that may be returned, on the meaning of uncertain or incidental findings, and on the procedures triggered by such results, is at least as important as information on the breadth of the disease panel. Materials should be linguistically and culturally adapted, and should make explicit space for the ethical questions raised by part of the parental population.

#### Consent frameworks.

In France, standard NBS operates on a non-opposition basis, with explicit written consent required only for the molecular component of cystic fibrosis screening and, since 2025, for spinal muscular atrophy. Most ongoing gNBS pilots, by contrast, have adopted opt-in frameworks (GUARDIAN, BabyScreen + , BabyDetect, Generation Study, PERIGENOMED). The differentiating role of ethical concerns, the explicit demands for confidentiality and legal safeguards, and the lower acceptability among parents in more vulnerable social positions point to dimensions that a mechanical transposition of the current non-opposition model would leave unanswered. Empirical work focused on parental information needs and consent preferences, including in the context of ongoing pilots, will be necessary to translate these stakes into operational design.

#### Governance and operational proportionality.

Sustaining parental acceptability will depend at least as much on governance as on technical performance. Data protection, clarity on analytic scope, procedures for residual bloodspots, and accessible channels for queries function as structural determinants of trust rather than peripheral safeguards. Involving parent and patient representatives in the development of information materials, consent procedures, and data-governance policies could strengthen ethical legitimacy and programme sustainability [38–41]. Operationally, parents supported genomic expansion when they perceived that benefits outweighed psychological and organisational burdens; maintaining this balance requires minimising additional steps, ensuring rapid confirmatory testing, and structuring support for the communication of uncertain results.

### Limitations and perspectives

This study has four main limitations. First, the design was prospective: parents evaluated hypothetical scenarios rather than making real-world decisions, and hypothetical framing may overestimate uptake. Stated intentions can nonetheless align with enrolment when options are concretely offered [25], and uptake in real-world gNBS pilots ranges widely (very low in BabySeq, very high in BabyDetect) depending on what is disclosed and how [57–61]. Prospective acceptability therefore functions as an anticipatory rather than predictive tool. Second, recruitment introduced selection bias. The two data-collection modalities (paper in maternity wards, online quota panel) broadened coverage but were not strictly comparable, and both required French literacy and digital access, likely under-representing non-French-speaking parents and families facing acute social vulnerability [17]. This probably leads to an underestimation of sociocultural gradients in acceptability. Third, our measurement strategy captured only part of the TFA structure. Affective attitude, perceived effectiveness, and ethicality were robustly measured, but burden, opportunity costs, and intervention coherence were not included as closed items (the latter being less salient in the French universal-care setting). Some concerns (non-invasiveness, confidentiality, legal safeguards) were captured only in free-text comments, whose limited volume (81 unique verbatims, 4.9% of respondents) constrained the depth of content analysis and prevented their integration into regression models; a dedicated qualitative study with a larger and more diversified sample would be needed to characterise these dimensions more systematically. Cost, which has emerged as a key driver in other settings [26,28,32,34], was not assessed. Although the TFA has been applied to other health interventions, our findings indicate that its core components remain relevant in a universal-care context, while further psychometric work is needed to confirm transferability. Fourth, the cross-sectional design did not capture how acceptability evolves over time, particularly across the transition from pregnancy to the postpartum period when absorptive capacity and psychological fatigue may alter decision-making [62]. Longitudinal designs embedded in implementation pilots will be needed to clarify how initial intentions are reframed once gNBS is concretely offered.

Several governance dimensions central to public and professional debates (criteria for inclusion, storage and reanalysis rules, secondary uses of data) were beyond the scope of this article. Preferences regarding treatability, onset, and actionability will be addressed separately, and divergences between parental and professional rationalities [56] warrant dedicated comparative analysis.

These limitations point to three directions for future work: expanding the sociocultural scope of research by including non-French-speaking parents and measuring constructs such as trust and health-system familiarity; clarifying how parents and professionals weigh competing rationalities around inclusion criteria, treatability, and ambiguous findings; and bridging prospective and real-world evidence by combining anticipatory surveys with longitudinal follow-up in pilots such as PERIGENOMED in France.

## Conclusions

Parental acceptability of eNBS and gNBS is high yet shaped by identifiable evaluative logics. Judgements rest primarily on anticipated benefits for the child and on a positive emotional orientation toward screening, while tolerance for uncertainty and ethical concerns differentiate assured from conditional support. Explicit reference to genetics moderates enthusiasm without reversing endorsement, indicating that gNBS is largely interpreted as an extension of an already valued programme. Sociodemographic and experiential factors modulate these orientations, with cultural distance and prior exposure to rare disease emerging as particularly relevant. The success of any expansion will therefore depend not only on technical performance but on proportionate, culturally adapted communication and on transparent governance. Real-world evidence from forthcoming pilots, including PERIGENOMED in France, will be essential for guiding responsible and socially legitimate adoption.

## Supporting information

S1 File Survey instrument. Full questionnaire administered to parents, including information sheets, consent procedures, sociodemographic items, acceptability measures, and evaluative components. Yellow highlights indicate online programming and skip logic; page breaks are marked.
(PDF)

S2 Table Site-level recruitment characteristics and completion rates (Population 1). Characteristics of participating maternity wards, recruitment periods, inclusion and non-inclusion reasons, and questionnaire completion metrics for the maternity-based recruitment (Population 1). * All multiple births recorded during the recruitment periods were twin births.
(PDF)

S3 Table Individual shifts in acceptability with mention of genetic testing and additional regression analyses. Cross-tabulations showing changes in individual acceptability judgments when genetic testing is explicitly mentioned, by sociodemographic characteristics, health literacy, and family situation. All observed associations were of small magnitude, with Cramer’s V values ranging from 0.06 to 0.12. Supplementary Table provides detailed cross-tabulations, cell counts, percentages, and exact p-values for all outcomes. This table summarizes the associations between respondent sociodemographic characteristics (rows) and the three main outcome variables (columns): general acceptability of expanded newborn screening, acceptability of expanded newborn screening with genetics, and individual opinion shift with mention of genetic testing. For each characteristic, cell values are presented as: n (% within characteristic) – that is, count and row percentage for each response category. Percentages are calculated excluding missing data. Totals for each subgroup are displayed in the first column. Total counts vary across variables due to missing data, as sociodemographic characteristics were not mandatory in the questionnaire. p-values (and Cramer’s V, if p < 0.1) are reported for each comparison, based on either Pearson’s Chi-squared or Fisher-Freeman-Halton (FFH) test according to expected cell counts (Cochran’s rule, simulation = 2000). Abbreviations: NBS: newborn screening, n: count, %: row percentage, Cramer’s V: effect size measure for categorical associations, ‡: FFH, *p < 0.1, **p < 0.05, ***p < 0.01, ***p < 0.001. This supplementary file also presents the best-fitting logistic regression models for parental acceptability outcomes, based on explanatory variables recoded as binary (agree or important vs not agree or not important). Models are reported for acceptability of eNBS and gNBS, using alternative outcome codings. Two specifications are shown: models including core acceptability components derived from the Theoretical Framework of Acceptability (affective attitude, perceived effectiveness, ethicality), and models estimated without this constraint. Model comparison relied on Akaike Information Criterion (AIC), variance inflation factors, and sample size. These analyses assess model robustness and the stability of associations across specifications.
(PDF)

S4 Table Themes expressed by parents regarding their choice of screening technique. This supplementary table presents free-text comments organised by thematic categories (rows) and by the screening technique criteria judged important by respondents (columns). This layout allows comparison of thematic patterns across technical preference profiles.
(PDF)

S5 Table Thematic analysis of comments on acceptability. S5a: Catalogue of verbatims classified by macro-theme and sub-theme, with definitions (n = 81 unique verbatims) Colours match the palette used in Fig 4. Sub-themes are listed in decreasing order of frequency. When a comment addressed several sub-themes, it was repeated and counted in each relevant category. S5b: Cross-tabulation of general and genomic acceptability among respondents who provided a free-text comment (n = 81 unique verbatims) Rows represent acceptability with genetic testing; columns represent acceptability in general. Each cell shows the count followed by the percentage of the comment subgroup. S5c: Full interpretation of qualitative material and mapping of parental profiles.
(PDF)

S6 Table Selection of published studies on parental attitudes toward newborn screening. Overview of selected published studies examining parental attitudes toward newborn screening and its expansion, including study design, population, and main findings.
(XLSX)

## Abbreviations

NBS: Newborn screening
eNBS: Expanded newborn screening
gNBS: Genomic newborn screening
TFA: Theoretical Framework of Acceptability
SeDeN: Séquençage et Dépistage Néonatal (national survey and research project)
CI: Confidence interval
OR: Odds ratio
AIC: Akaike Information Criterion
VIF: Variance inflation factor
