# Supplementary material for: Parental acceptability of newborn screening expansion in the genomic era: A nationwide French survey informed by the Theoretical Framework of Acceptability (SeDeN-p3)
Source: PLoS One. 2026 Jun 15;21(6):e0343754. doi: 10.1371/journal.pone.0343754 (PMC13268192; doi:10.1371/journal.pone.0343754)
Supplement: S1 File — Full questionnaire administered to parents, including information sheets, consent procedures, sociodemographic items, acceptability measures, and evaluative components. Yellow highlights indicate online programming and skip logic; page breaks are marked. (PDF) [file pone.0343754.s001.pdf]

Supplementary material S1

Yellow highlights indicate online programming elements / skip logic ; A line indicates a page break
En jaune : Programmation en ligne / Filtres ; un trait indique un changement de page

---

Thank you for clicking on our survey. / *Merci d'avoir cliqué sur notre enquête.*

Our multidisciplinary research team [http://www.translad.org/presentation.html] is investigating the role of new technologies in the screening of certain rare diseases at birth (newborn screening).

Notre groupe de chercheurs [http://www.translad.org/presentation.html] de différentes disciplines travaille sur la place des nouvelles technologies dans le dépistage de certaines maladies rares à la naissance (dépistage néonatal).

Your responses to this questionnaire will help us identify the topics that matter most to parents regarding newborn screening in France. All answers will remain completely anonymous and will be used solely for scientific research *purposes.*

Vos réponses à ce questionnaire aideront à comprendre les sujets importants pour les parents autour du dépistage néonatal en France. Elles resteront totalement anonymes et ne seront utilisées qu'à des fins de recherche scientifique.

There are no right or wrong answers, and choosing to complete—or not complete—this questionnaire will have no impact on the care you or your child(ren) receive. Nevertheless, your opinion is important for the future development of newborn screening in the coming years.

Il n'y a pas de bonnes ou de mauvaises réponses et répondre à ce questionnaire (ou non) n'aura aucun impact sur votre prise en charge ou celle de votre (vos) enfant(s). Votre avis est néanmoins important à prendre en compte pour l'évolution du dépistage néonatal dans les prochaines années.

We are surveying healthcare professionals, policy-makers (public authorities and influential organizations), and parents on this topic.

Nous interrogeons les professionnels de santé, les décideurs (publics et groupes influents) et les parents à ce sujet.

The questionnaire should take approximately 20 minutes to complete.

Le remplissage du questionnaire devrait vous demander environ 20 minutes.

If you have any questions, please feel free to contact the study coordinator at camille.level@chu-dijon.fr.

Si vous souhaitez des précisions, n'hésitez pas à contacter le coordonnateur de l'étude à camille.level@chu-dijon.fr

TRANSLAD Médecine translationnelle dans les anomalies du développement — Société Française de Dépistage Néonatal — CHU Dijon Bourgogne — SeDeN-p3

Your unique identifier/*Votre identifiant*

We will begin by creating your unique identifier/ *Nous allons commencer par créer votre identifiant*

Your responses to this questionnaire are anonymous. To exercise your rights of access, correction, objection, or deletion of your data, you will need to provide this identifier so that we can retrieve your answers. Please keep it in a safe place.
Vos réponses à ce questionnaire sont anonymes. Pour pouvoir exercer vos droits d'accès, de rectification, d'opposition ou de suppression de vos données, vous devrez nous communiquer l'identifiant ci-dessous pour que nous puissions retrouver vos réponses. Vous devez donc conserver cet identifiant.

You may record this identifier on the information sheet you received (either in paper form or downloadable here: [Information Sheet]). This document provides details about the study's objectives and how your data will be processed. We strongly encourage you to read it.
Vous pouvez le noter sur la note d'information qui vous a été remise en version papier ou téléchargeable ici. [Note d'information]. Cette note vous apporte des informations sur l'étude, ses objectifs et sur le traitement qui sera fait à vos données. Nous vous invitons fortement à la lire.

Your identifier is: Q___/___/___/___/ **(random alphanumeric code; duplicates prevented)**
Voici votre identifiant : Q___/___/___/___/ *Code alphanumérique aléatoire et empêcher les doublons*

By proceeding to the next page, you acknowledge that you have read the information sheet and agree to its terms.To begin the questionnaire, please click the "Next" button below.
En passant à la page suivante, vous reconnaissez avoir lu cette note d'information et attestez en accepter les conditions. Pour débuter le questionnaire, merci de cliquer sur le bouton « Suivant » ci-dessous.

Your profile/*Votre profil*

To begin, please answer a few questions about yourself / *Pour débuter, quelques questions sur vous-même.*

What is your age? / *Quel est votre âge ?*
Please enter a whole number only (no decimals). / Merci d'indiquer uniquement un nombre entier, sans virgule.
/___/ years / ans PROG : integer only; min: 18, max: 50 ; nombre entier uniquement / min : 18 – max : 50

What is the age of your youngest child? / *Quel est l'âge de votre plus jeune enfant ?*

- Less than 1 week / *Moins de 1 semaine*
- Between 8 days and 6 months / *Entre 8 jours et 6 mois*
- Between 6 and 12 months / *Entre 6 et 12 mois*
- Between 12 and 24 months (1–2 years) / *Entre 12 et 24 mois (1 à 2 ans)*
- Between 24 and 36 months (2–3 years) / *Entre 24 et 36 mois (2 à 3 ans)*
- More than 36 months (over 3 years) / *Plus de 36 mois (plus de 3 ans)* ***→ Exclusion***

If "Less than 1 week" is selected/Si répond 'Moins de 1 semaine'

In which maternity ward was your child born? / *Dans quelle maternité est-il né ?*

/____/

Please do not enter any personal or identifying information. / Merci de veiller à ne renseigner aucune donnée personnelle ou identifiante dans cette zone de texte.

What is your highest completed diploma? / *Quel est votre diplôme le plus élevé ?*

- No diploma / *Aucun diplôme*
- CEP, BEPC, elementary brevet, collège brevet, DNB / *CEP, BEPC, brevet élémentaire, brevet des collèges, DNB*
- CAP, BEP, or equivalent vocational diploma / *CAP, BEP ou diplôme de niveau équivalent*
- General or technological baccalauréat, higher brevet, law capacity certificate, DAEU, ESEU / *Baccalauréat général ou technologique, brevet supérieur, capacité en droit, DAEU, ESEU*
- Professional baccalauréat, professional brevet, technician's diploma or equivalent / *Baccalauréat professionnel, brevet professionnel, de technicien ou d'enseignement, diplôme équivalent*
- BTS, DUT, DEUG, DEUST, health or social work diploma at Bac+2 level, or equivalent / *BTS, DUT, DEUG, DEUST, diplôme de la santé ou du social de niveau bac+2, diplôme équivalent*
- Licence, professional licence, maîtrise, or equivalent (Bac+3 or Bac+4) / *Licence, licence pro, maîtrise, diplôme équivalent de niveau bac+3 ou bac+4*
- Master, DEA, DESS, grande école diploma (Bac+5), health doctorate / *Master, DEA, DESS, diplôme grande école niveau bac+5, doctorat de santé*
- Research doctorate (non-health) / *Doctorat de recherche (hors santé)*

Recode into three categories/*Puis RECODE en 3*

Please enter your postal code and select your municipality / *Merci de taper le code postal de votre habitation et sélectionner votre commune*
PROG : Dropdown menu; display town(s) matching the postal code; then recode to urban area/region
Menu déroulant / Afficher la ou les villes qui correspondent au code postal puis RECODE Agglomération / Région

Newborn screening / *Le dépistage néonatal*

Have you previously received any information about newborn screening before participating in this study?
Avez-vous déjà eu des informations sur le dépistage néonatal, avant votre participation à cette étude ?

- Yes / *Oui*
- No / *Non*

Newborn screening as currently performed in France / *Le dépistage néonatal, actuellement en France*

Below is information on how newborn screening is conducted in France today.
Voici quelques informations sur le dépistage néonatal tel qu'il est actuellement réalisé en France.

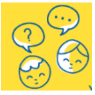

In France, newborn screening (the Guthrie test) is offered free of charge to all infants on day 3 of life. It detects certain rare diseases as early as possible, allowing treatment or care to begin before any symptoms appear. The goal is to prevent permanent damage and enable the child to develop and grow optimally.

En France, un dépistage est proposé gratuitement à tous les bébés au 3ème jour de vie : il s'agit du dépistage néonatal ou Test de Guthrie. Il permet de détecter le plus tôt possible certaines maladies rares et de commencer un traitement ou une prise en charge avant même que des signes de la maladie n'apparaissent. Le but est d'éviter que le bébé ait des séquelles définitives et de lui permettre de se développer et de grandir le mieux possible.

Screening is free and systematically offered, but parental consent is required.
Ce dépistage est gratuit et il est systématiquement proposé, mais l'accord des parents est obligatoire.

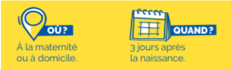

In practice, a midwife or nurse collects a few drops of blood from the baby three days after birth and places them on filter paper. [Image: Guthrie test sample collection]
Actuellement, en pratique, une sage-femme ou une infirmière recueille quelques gouttes de sang au bébé 3 jours après la naissance et les dépose sur un papier buvard. [PROG : https://static.cnsf.asso.fr/wp-content/uploads/2020/03/Guthrie.jpg]

This test is quick and safe. The filter paper is then sent to a laboratory for analysis.
Ce test est rapide et sans danger. Le papier buvard est ensuite envoyé dans un laboratoire pour être analysé.

If no disease is detected, parents receive no further contact. If a disease is detected, the family is summoned by a physician within 3–4 weeks for confirmatory testing and to receive the results, an explanation of the condition, and information on follow-up.
Quand aucune maladie n'est trouvée, les parents ne sont pas recontactés.
À l'inverse, si une maladie est détectée, ils sont convoqués par un médecin sous 3 à 4 semaines maximum, afin de faire des tests complémentaires et/ou leur transmettre le résultat et leur expliquer la maladie et son suivi.

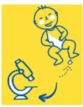

The screened diseases are rare but serious—often genetic—and lifelong. They include:
- **Certain hormonal disorders: hormone dysfunction leading to significant sequelae (intellectual disability, growth retardation, etc.).**
- **Certain metabolic disorders: impaired nutrient metabolism causing serious outcomes (intellectual disability, growth delay, etc.).**
- **Cystic fibrosis: frequent lung infections, respiratory difficulties, and digestive problems.**
- **Sickle-cell disease (in infants whose parents originate from high-risk regions): fragile, rigid red blood cells causing anemia, pain crises, and infections.**

Les maladies dépistées sont rares, mais elles sont graves, souvent d'origine génétique et durent toute la vie. Il s'agit de :
- *Certaines maladies hormonales : les hormones ne fonctionnent pas correctement et provoquent des séquelles importantes (déficit intellectuel, retard de croissance...).*
- *Certaines maladies métaboliques : la bonne utilisation des aliments dans l'organisme est empêchée, ce qui peut entraîner des séquelles importantes (déficit intellectuel, retard de croissance…).*
- *La mucoviscidose : des infections fréquentes aux poumons et une gêne pour respirer sont présentes, ainsi que des difficultés à digérer.*
- *La drépanocytose (chez les bébés dont les parents sont originaires de régions à risque) : les globules rouges sont fragiles et rigides, ce qui conduit à une anémie (manque de globules rouges dans le sang), des douleurs et des infections.*

For a complete list of currently screened conditions, see / *Plus d'informations sur les maladies dépistées actuellement à la naissance ici [https://depistage-neonatal.fr/les-maladies-depistees/]*

A separate newborn hearing screening is also performed; this auditory test does not use filter paper.
Il existe également un dépistage de la surdité à la naissance. Ce test auditif n'est pas réalisé à partir du papier buvard.

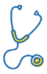

If a baby is diagnosed with one of these conditions, a specialized medical team implements appropriate follow-up care and treatment—such as medication, disease-specific dietary management, or other therapies—to support the child's optimal development.

Si le bébé est atteint d'une des maladies, un suivi médical est mis en place par une équipe spécialisée avec plusieurs types de traitements possibles (des médicaments, une alimentation adaptée à la maladie, ou d'autres méthodes).
Ces traitements ou prises en charge permettront au bébé de se développer au mieux.

These details are adapted from the leaflet "Le dépistage dès la naissance, c'est important" published by the Haute Autorité de Santé, the Centre National de Coordination du Dépistage Néonatal, and the Ministry of Solidarity and Health.
Ces informations sont issues du dépliant "Le dépistage dès la naissance, c'est important" [PROG : https://depistage-neonatal.fr/wp-content/uploads/2021/11/Dépliant-DNN.pdf] de la Haute Autorité de Santé, du Centre National de Coordination du Dépistage Néonatal et du Ministère des Solidarités et de la Santé.

The expansion of newborn screening / *L'extension du dépistage néonatal*

The number of conditions screened varies widely between countries. In France, 7 disorders are currently screened in newborns (presented above), and 7 additional metabolic diseases will soon be added (leucinosis, homocystinuria, tyrosinemia type 1, glutaric aciduria type 1, isovaleric acidemia, long-chain hydroxyacyl-CoA dehydrogenase deficiency, and carnitine uptake deficiency). This remains fewer than in many other developed countries.
Le nombre de maladies dépistées est très variable d'un pays à l'autre. En France, on dépiste actuellement 7 maladies chez les bébés à la naissance (présentées précédemment) et 7 autres maladies métaboliques seront bientôt introduites dans le dépistage néonatal (la leucinose, l'homocystinurie, la tyrosinémie de type 1, l'acidurie glutarique de type 1, l'acidurie isovalérique, le déficit en déshydrogénase des hydroxyacyl-CoA de chaîne longue, et le déficit en captation de carnitine).
Cela est globalement moins que dans d'autres pays développés.
[https://www.parents.fr/actualites/bebe/depistage-neonatal-bientot-elargi-a-7-autres-maladies-431819]

For the remainder of this questionnaire, we ask your opinion on expanding French newborn screening. This is a hypothetical scenario: please distinguish these questions from current screening practice.
Pour la suite de ce questionnaire, nous vous demandons votre avis sur l'extension du dépistage néonatal français. Il s'agit d'une ***situation non réelle****, pour laquelle nous vous demandons d'imaginer ce que vous souhaiteriez, à bien distinguer du dépistage néonatal actuel.*

> PROG: **The numbers correspond to the weights assigned to each response option for the quantitative analysis of the scales**. / Les chiffres correspondent au poids des modalités pour l'analyse quantitative des échelles.

Screening more conditions at birth for my baby seems to me…
Dépister plus de maladies à la naissance chez mon bébé me paraît …

1. Completely unacceptable / *Complètement inacceptable*
2. Somewhat unacceptable / *Plutôt inacceptable*
3. No opinion / *Pas d'opinion*
4. Somewhat acceptable / *Plutôt acceptable*
5. Completely acceptable / *Tout à fait acceptable*

Please indicate your level of agreement with the following statements:
Donnez votre avis sur les propositions suivantes :

Screening more conditions at birth for my baby can improve their health TFA - Perceived efficacy
Dépister plus de maladies à la naissance chez mon bébé peut améliorer sa santé

1. Strongly disagree / *Pas du tout d'accord*
2. Somewhat disagree / *Plutôt pas d'accord*
3. No opinion / *Pas d'opinion*
4. Somewhat agree / *Plutôt d'accord*
5. Strongly agree / *Tout à fait d'accord*

I would like more conditions to be screened at birth for my baby TFA - Attitude affective
J'aimerais que l'on dépiste plus de maladies à la naissance chez mon bébé

1. Strongly disagree / *Pas du tout d'accord*
2. Somewhat disagree / *Plutôt pas d'accord*
3. No opinion / *Pas d'opinion*
4. Somewhat agree / *Plutôt d'accord*
5. Strongly agree / *Tout à fait d'accord*

Screening more conditions at birth for my baby raises moral or ethical concerns for me TFA - Ethicality
Dépister plus de maladies à la naissance chez mon bébé me pose des questions morales ou éthiques

1. Strongly disagree / *Pas du tout d'accord*
2. Somewhat disagree / *Plutôt pas d'accord*
3. No opinion / *Pas d'opinion*
4. Somewhat agree / *Plutôt d'accord*
5. Strongly agree / *Tout à fait d'accord*

The expansion of newborn screening / *L'extension du dépistage néonatal*

The expansion of newborn screening also involves considerations about the techniques used.
L'extension du dépistage néonatal inclut également des réflexions sur les techniques à utiliser.

For example, some conditions can only be detected using a genetic test. In this case, no additional blood draw would be required in the maternity ward. The baby's blood would still be collected on filter paper as usual, but a genetic analysis would then be applied.
Par exemple, certaines maladies ne peuvent être dépistées qu'à l'aide d'un test génétique.
Il n'y aurait pas besoin de prélèvement de sang en plus à la maternité dans ce cas-là. Le sang du bébé serait toujours récupéré sur un papier buvard de la même façon, mais un test génétique serait ensuite utilisé dans les analyses.

Screening more conditions at birth using genetic tests (DNA analysis) seems to me…

Dépister plus de maladies à la naissance chez mon bébé, à l'aide de tests génétiques (analyse de l'ADN), me paraît …

1. Completely unacceptable / *Complètement inacceptable*
2. Somewhat unacceptable / *Plutôt inacceptable*
3. No opinion / *Pas d'opinion*
4. Somewhat acceptable / *Plutôt acceptable*
5. Completely acceptable / *Tout à fait acceptable*

You may elaborate on your response if you wish: */ Vous pouvez préciser votre réponse si vous le souhaitez :*

/___________/

Please do not enter any personal or identifying information in this text box. */ Merci de veiller à ne renseigner aucune donnée personnelle ou identifiante dans cette zone de texte.*

The expansion of newborn screening / *L'extension du dépistage néonatal*

The number of conditions that can be screened and the risk of uncertain results depend on the technique employed.
Le nombre de maladies que l'on peut dépister et le risque de résultats incertains dépendent du type de technique utilisée.

Below are three different techniques, varying in the number of conditions screened and in the risk of uncertain outcomes:
Nous vous présentons ci-dessous 3 techniques différentes qui varient en fonction du nombre de maladies qu'elles peuvent dépister, mais aussi en fonction du risque de résultats incertains.

Technique A

Conditions screened: Many / *Nombre de maladies dépistées : Beaucoup*
Risk of uncertain results: High / *Risque de résultats incertains : Élevé*

Technique B

Conditions screened: Moderate / *Nombre de maladies dépistées : Moyen*
Risk of uncertain results: Moderate / *Risque de résultats incertains : Moyen*

Technique C

Conditions screened: Few / *Nombre de maladies dépistées : Peu*
Risk of uncertain results: Low / *Risque de résultats incertains : Faible*

In summary, Technique A screens the most conditions but carries a high risk of uncertainty; Technique C screens the fewest conditions with mostly reliable results; Technique B is an intermediate option, it screens

a moderate number of conditions and carries a moderate risk of uncertain results, falling between Techniques A and C.

En résumé, la technique A permet de dépister beaucoup de maladies, mais comporte un risque élevé de résultats incertains.
Au contraire, la technique C permet de dépister peu de maladies, mais les résultats sont en grande partie sûrs. La technique B est un intermédiaire, elle dépiste un nombre moyen de maladies et présente un risque de résultats incertains situé entre celui de la technique A et celui de la C.

For screening one condition in my baby at birth, I prefer to use…
Pour dépister une maladie chez mon bébé à la naissance, je préfère qu'on utilise la …

- Technique A
- Technique B
- Technique C

Which factor influenced your choice the most? / *Quelle dimension a le plus pesé dans votre choix ?*

- The number of conditions screened / *Le nombre de maladies dépistées*
- The risk of uncertain results / *Le risque de résultats incertains*
- Both factors were equally important in my decision / *Les deux ont pesé de la même façon dans ma décision*

You may elaborate on your response if you wish: */ Vous pouvez préciser votre réponse si vous le souhaitez :*

/___________/

Please do not enter any personal or identifying information in this text box./Merci de veiller à ne renseigner aucune donnée personnelle ou identifiante dans cette zone de texte.

Getting to know you better */ Mieux vous connaître*

The questionnaire is almost complete. / *Le questionnaire touche à sa fin.*

Other studies have shown that certain characteristics (age, occupation, income, experience with genetic testing and/or rare diseases, etc.) can influence your opinion on newborn screening. Thank you for completing these final questions to help us better get to know you and understand your choices.

D'autres études ont montré que certaines caractéristiques (âge, métier, revenus, expérience sur les tests génétiques et/ou maladies rares, etc.) peuvent influencer votre avis le dépistage néonatal. Merci de remplir ces dernières questions pour nous permettre de mieux vous connaître et comprendre vos choix.

You may, if you wish, indicate that you prefer not to answer any question.

Vous pouvez, si vous le souhaitez, préciser que vous préférez ne pas y répondre.
Responses of "I prefer not to answer" were recoded as missing (NA)/Recode : si « Je préfère ne pas répondre » = NA

Are you or a close family member affected by a rare disease?
Vous ou un des membres de votre famille proche, êtes-vous concerné par une maladie rare ?

- Yes / *Oui*
- No / *Non*
- I'm not sure it qualifies as a rare disease / *Je ne suis pas sûr(e) qu'il s'agisse d'une maladie rare*
- I don't know / *Je ne sais pas*
- I prefer not to answer / *Je préfère ne pas répondre*

RECODE : "Yes" responses recoded as Concerned, others as Not concerned / Oui = Concerné, le reste = Non concerné

You identify as…
Vous vous identifiez plus à …

- A man / Un homme
- A woman / Une femme
- Other / Autre
- I prefer not to answer / *Je préfère ne pas répondre*

In which country were you born?
Dans quel pays êtes-vous né(e) ? /___________/
You are not required to answer this question. Please do not enter any other personal or identifying information in this text box.

Vous n'êtes pas obligé(e) de répondre à cette question. Merci de veiller à ne renseigner aucune autre donnée personnelle ou identifiante dans cette zone de texte.

What is your marital status?

Quelle est votre situation matrimoniale ?

- Single / *Célibataire*
- Married or in a civil partnership / *Marié(e), pacsé(e)*
- In a free union or cohabiting / *Union libre, concubinage*
- Divorced or separated / *Divorcé(e), séparé(e)*
- Widowed / *Veuf, veuve*
- I prefer not to answer / *Je préfère ne pas répondre*

Recode marital status as follows: In a couple: Married or in a civil partnership; In a free union or cohabiting AND Single parent: Single; Divorced/Separated; Widowed / *RECODE : En couple : Marié(e), Pacsé(e), Union libre / Concubinage ET Parent isolé : Célibataire, Divorcé(e) / Séparé(e), Veuf / Veuve*

How many children do you have or have had?
Combien d'enfants avez-vous ou avez-vous eu ?
You are not required to answer this question. */ Vous n'êtes pas obligé(e) de répondre à cette question.*
If you are completing this questionnaire in the maternity ward, include your newborn. */ Si vous remplissez le questionnaire à la maternité, celui qui vient de naître compris.*
/_____/ child(ren) / *enfant(s)* PROG : interger, min: 1 – max: 30

How many people (adults and children), including yourself, live in your household?
Combien de personnes (adultes et enfants), y compris vous-même, vivent dans votre foyer ?

- 1 person / *1 personne*
- 2 people / *2 personnes*
- 3 people / *3 personnes*
- 4 people / *4 personnes*
- 5 people or more / *5 personnes ou plus*
- I prefer not to answer / *Je préfère ne pas répondre*

In which professional sector do you work?
Dans quel secteur professionnel travaillez-vous ?

- Agriculture, Agri-food, Environment / *Agriculture, Agroalimentaire, Environnement*
- Arts, Culture / *Arts, Culture*
- Construction, Public Works / *Bâtiment, Travaux publics*
- Business, Management, Economics / *Commerce, Gestion, Économie, Management*
- Law, Political Science, Security / *Droit, Sciences politiques, Sécurité*
- Education, Training / *Enseignement, Formation*
- Hospitality, Catering, Tourism / *Hôtellerie, Restauration, Tourisme*
- Industry / *Industrie*
- Information, Communication / *Information, Communication*
- Literature, Languages, Humanities / *Lettres, Langues, Sciences humaines*
- Health / *Santé*
- Sciences / *Sciences*
- Social Services / *Social*
- Sports, Recreation / *Sport, Animation*
- Transportation, Logistics / *Transports, Logistique*
- Other / *Autre*
- I prefer not to answer / *Je préfère ne pas répondre*

If "Other," please specify / *Si « Autre », merci de préciser*
/________/

Please do not enter any other personal or identifying information in this text box. / *Merci de veiller à ne renseigner aucune autre donnée personnelle ou identifiante dans cette zone de texte.*

Regarding your household finances, would you describe your situation as…?
Concernant les finances de votre foyer, vous sentez-vous … ?

- Comfortable / *À l'aise*
- Balanced / *À l'équilibre*
- Needs monitoring / *À surveiller*
- Struggling / *En difficulté*
- I prefer not to answer / *Je préfère ne pas répondre*

Having completed this questionnaire, you have the right to be informed of the overall results of this study. Would you like to receive these results?
Ayant répondu à ce questionnaire, vous avez le droit d'être informé des résultats généraux de cette étude. Souhaitez-vous être tenu au courant des résultats de l'étude ?

- Yes / *Oui*
- No / *Non*

PROG **If Yes** / *Si a répondu Oui* :

PROG **If contacted in maternity ward** / *Si le parent a été contacté à la maternité*
Please give your email address to the person or team who introduced the study to you in the maternity ward.
Veuillez donner votre adresse mail à la personne ou à l'équipe qui vous a présenté l'étude à la maternité.

PROG **If respond as part of the survey panel** / *Si a répondu en tant que membre du panel de l'institut de sondage*
Please enter your email address: / *Merci de renseigner votre adresse mail*
/__________________/

Your email will be used solely to send you the study results. Please do not enter any other personal or identifying information in this field. */ Votre adresse mail ne sera utilisée que pour vous informer des résultats de cette enquête. Merci de ne renseigner aucune autre donnée personnelle ou identifiante dans cette zone de texte.*
PROG : **Email is required; store email separately from survey responses to maintain anonymity.** / @ obligatoire / stocker l'adresse mail dans une base de données différente de celle avec les réponses aux questions pour éviter de faire le chaînage.

To submit your questionnaire, click the button below. / *Pour valider votre questionnaire, cliquez sur le bouton ci-dessous.*

This questionnaire is now complete. The TRANSLAD University-Hospital Federation thanks you for participating in this survey. */ Ce questionnaire est à présent terminé. La Fédération Hospitalo-Universitaire TRANSLAD vous remercie d'avoir participé à son enquête.*

This questionnaire may have caused you some distress. If you need support, a psychological resource is available to speak with you.
Ce questionnaire peut vous avoir angoissé. Si vous en avez besoin, une ressource psychologique est disponible pour échanger avec vous.
PROG **If contacted in maternity ward** / Si le parent a été contacté à la maternité
Please inform the healthcare professional who introduced the study to you.
Si tel est le cas, merci d'en faire part au professionnel de santé qui vous a présenté l'étude à la maternité.

PROG **If respond as part of the survey panel** / Si a répondu en tant que membre du panel de l'institut de sondage
Please contact us at camille.level@chu-dijon.fr
Si tel est le cas, merci de nous contacter à camille.level@chu-dijon.fr
If you have any questions about this questionnaire or simply wish to discuss the study, do not hesitate to contact the study coordinator at camille.level@chu-dijon.fr.
Si le remplissage de ce questionnaire vous pose question ou si vous souhaitez tout simplement discuter de l'étude, n'hésitez pas à contacter le coordonnateur de l'étude à camille.level@chu-dijon.fr

For more information on current newborn screening, you may consult:
Voici quelques ressources si vous souhaitez en savoir plus sur le dépistage néonatal actuel :
https://depistage-neonatal.fr/
https://www.maladiesraresinfo.org/
Le dépistage dès la naissance, c'est important

Thank you for your participation!
Nous vous remercions pour votre participation !
