# Supplementary material for: Parental acceptability of newborn screening expansion in the genomic era: A nationwide French survey informed by the Theoretical Framework of Acceptability (SeDeN-p3)
Source: PLoS One. 2026 Jun 15;21(6):e0343754. doi: 10.1371/journal.pone.0343754 (PMC13268192; doi:10.1371/journal.pone.0343754)
Supplement: S2 Table — Characteristics of participating maternity wards, recruitment periods, inclusion and non-inclusion reasons, and questionnaire completion metrics for the maternity-based recruitment (Population 1). * All multiple births recorded during the recruitment periods were twin births. (PDF) [file pone.0343754.s002.pdf]

Supplementary material S2

	CLAMART	DIJON	PARIS NECKER	VESOUL	TOTAL
**Recruitment characteristics**					
**Site selection criteria and maternity unit characteristics (2022)**					
Region	Île-de-France	Bourgogne-Franche-Comté	Île-de-France	Bourgogne-Franche-Comté	-
Department	Hauts-de-Seine (92)	Côte-d'Or (21)	Paris (75)	Haute-Saône (70)	
Urbanization level	Densely peri-urban	Intermediate urban	Capital metropolis	Rural	-
Level of maternity care	Level 3	Level 3	Level 3	Level 2	-
Average daily deliveries	9 (3,434/365)	9 (3,114/365)	8 (3,045/365)	3 (998/365)	-
Recruitment period (First Patient In – Last Patient In)	3 October 2022 – 9 October 2022	9 September 2022 – 23 September 2022	3 October 2022 – 9 October 2022	3 October 2022 – 30 November 2022	-
Actual recruitment days (days with on-site recruitment activity)	7	13	7	27	54
**Total number of deliveries during the recruitment period**	**58**	**122**	**54**	**139**	**373**
Singleton births with a live newborn at the end of the period	50	111	46	135	**342**
*Twin births, counted as one family per twin pair**	5	6	5	2	**18**
TOP, stillbirths, or fetal deaths	3	5	3	2	**13**
**Reasons for non-inclusion**					
**Non-eligible population**					
Language barrier	13	17	42	1	**73**
Early discharges	10		6		**16**
Over-age infants	6	5	4		**15**
Vulnerable situations	4	2			**6**
Cognitive barrier	3		2		**5**
Over-age parent		1			**1**
Reason not reported		2	3		**77**
**Non-enrolled population**					
Refusals (before or after study information)	24	14	2	3	**43**
Questionnaires finally not returned	9	12	7		**28**
Transfer to another maternity ward/unit	4	3	9		**16**
**Questionnaire data**					
**Number of questionnaires collected** (including complete questionnaires)	121 (115)	125 (112)	72 (70)	96 (95)	**414 (392)**
Online	35 (32)	43 (34)	0 (0)	3 (3)	**81 (69)**
Paper	86 (83)	82 (78)	72 (70)	93 (92)	**333 (323)**
**Average number of questionnaires collected per day of recruitment**	17 (121/7)	10 (125/13)	10 (72/7)	4 (96/27)	8 (414/54)
**TOTAL COMPLETION RATE**	**95,0% (115/121)**	**89,6% (112/125)**	**97,2% (70/72)**	**99,0% (95/96)**	**94,7% (392/414)**
Online completion rate	91,4% (32/35)	79,1% (34/43)	N/A	100% (3/3)	**85,2% (69/81)**
Paper completion rate	96,5% (83/86)	95,1% (78/82)	97,2% (70/72)	99,0% (92/93)	**97,0% (323/333)**
**Percentage of complete questionnaires filled out by a female respondent**	55,7% (64/115)	67,0% (75/112)	57,1% (40/70)	50,5% (48/95)	**57,9% (227/392)**
**Non-analysis population**					
Incomplete questionnaires (<50%)	5	11			**16**
Over-age parents	1		2	1	**4**
Over-age infants		2			**2**
