# Supplementary material for: Parental acceptability of newborn screening expansion in the genomic era: A nationwide French survey informed by the Theoretical Framework of Acceptability (SeDeN-p3)
Source: PLoS One. 2026 Jun 15;21(6):e0343754. doi: 10.1371/journal.pone.0343754 (PMC13268192; doi:10.1371/journal.pone.0343754)
Supplement: S3 Table — Cross-tabulations showing changes in individual acceptability judgments when genetic testing is explicitly mentioned, by sociodemographic characteristics, health literacy, and family situation. All observed associations were of small magnitude, with Cramer’s V values ranging from 0.06 to 0.12. Supplementary Table provides detailed cross-tabulations, cell counts, percentages, and exact p-values for all outcomes. This table summarizes the associations between respondent sociodemographic characteristics (rows) and the three main outcome variables (columns): general acceptability of expanded newborn screening, acceptability of expanded newborn screening with genetics, and individual opinion shift with mention of genetic testing. For each characteristic, cell values are presented as: n (% within characteristic) – that is, count and row percentage for each response category. Percentages are calculated excluding missing data. Totals for each subgroup are displayed in the first column. Total counts vary across variables due to missing data, as sociodemographic characteristics were not mandatory in the questionnaire. p-values (and Cramer’s V, if p < 0.1) are reported for each comparison, based on either Pearson’s Chi-squared or Fisher-Freeman-Halton (FFH) test according to expected cell counts (Cochran’s rule, simulation = 2000). Abbreviations: NBS: newborn screening, n: count, %: row percentage, Cramer’s V: effect size measure for categorical associations, ‡: FFH, *p < 0.1, **p < 0.05, ***p < 0.01, ***p < 0.001. This supplementary file also presents the best-fitting logistic regression models for parental acceptability outcomes, based on explanatory variables recoded as binary (agree or important vs not agree or not important). Models are reported for acceptability of eNBS and gNBS, using alternative outcome codings. Two specifications are shown: models including core acceptability components derived from the Theoretical Framework of Acceptability (affective attitude, perce [file pone.0343754.s003.pdf]

Supplementary material S3

Characteristic	Total[1]	Screening my baby for more diseases at birth seems to me ...					Screening my baby for more diseases at birth, using genetic tests (DNA analysis), seems to me …					Individual opinion shift with mention of genetic testing		
		Completely unacceptable	Unacceptable	No opinion	Acceptable	Completely acceptable	Completely unacceptable	Unacceptable	No opinion	Acceptable	Completely acceptable	Negatif shift	No shift	Positive shift
**Characteristic**	**Total[1]**	N = 25	N = 51	N = 34	N = 355	N = 1,166	N = 33	N = 91	N = 44	N = 565	N = 898	N = **443**	**N = 1,047**	**N = 141**
**INDIVIDUAL CHARACTERISTICS**														
Gender of respondent	***100.0% (1,608)***	1.6% (25)	3.2% (51)	1.7% (27)	21.6% (347)	72.0% (1,158)	2.0% (32)	5.5% (88)	2.3% (37)	34.7% (558)	55.5% (893)	27.0% (434)	64.3% (1,034)	8.6% (139)
Female	100.0% (1,129)	1.2% (13)	2.6% (29)	1.6% (18)	20.8% (235)	73.9% (834)	1.9% (22)	5.0% (57)	2.2% (25)	35.7% (403)	55.1% (622)	28.9% (326)	63.1% (712)	8.0% (90)
Male	100.0% (479)	2.5% (12)	4.6% (22)	1.9% (9)	23.4% (112)	67.6% (324)	2.1% (10)	6.5% (31)	2.5% (12)	32.4% (155)	56.6% (271)	22.5% (108)	67.2% (322)	10.2% (49)
**p-value**		**0.023** (0.08)**					0.625					**0.019** (0.07)**		
Country of birth	***100.0% (1,506)***	1.5% (22)	2.9% (44)	1.6% (24)	21.4% (322)	72.6% (1,094)	1.9% (29)	5.3% (80)	2.3% (35)	34.0% (512)	56.4% (850)	26.6% (401)	65.0% (979)	8.3% (125)
France	100.0% (1,373)	1.4% (19)	2.6% (36)	1.5% (20)	20.6% (283)	73.9% (1,015)	1.7% (23)	5.0% (69)	2.0% (28)	33.8% (464)	57.5% (789)	26.4% (362)	65.4% (897)	8.2% (113)
Foreign country	100.0% (133)	2.3% (3)	6.0% (8)	3.0% (4)	29.3% (39)	59.4% (79)	4.5% (6)	8.3% (11)	5.3% (7)	36.1% (48)	45.9% (61)	29.3% (39)	61.7% (82)	9.0% (12)
**p-value**		**0.003*** (0.10)[‡]**					**0.003*** (0.10)**					0.690		
Age category of respondent	***100.0% (1,634)***	1.5% (25)	3.1% (51)	2.0% (33)	21.8% (356)	71.5% (1,169)	2.0% (33)	5.6% (92)	2.7% (44)	34.6% (564)	55.0% (897)	27.1% (442)	64.2% (1,045)	8.7% (141)
< 25 years	100.0% (89)	2.2% (2)	7.9% (7)	2.2% (2)	19.1% (17)	68.5% (61)	2.3% (2)	12.5% (11)	3.4% (3)	40.9% (36)	40.9% (36)	32.2% (28)	62.1% (54)	5.7% (5)
25-35 years	100.0% (805)	1.0% (8)	3.2% (26)	1.9% (15)	21.2% (171)	72.7% (585)	2.0% (16)	5.8% (47)	2.6% (21)	37.0% (298)	52.5% (423)	29.9% (240)	62.2% (500)	8.0% (64)
> 35 years	100.0% (740)	2.0% (15)	2.4% (18)	2.2% (16)	22.7% (168)	70.7% (523)	2.0% (15)	4.6% (34)	2.7% (20)	31.2% (230)	59.4% (438)	23.6% (174)	66.6% (491)	9.8% (72)
**p-value**		0.161					**0.009*** (0.08)**					**0.041** (0.06)**		
**HEALTH LITERACY AND PRIOR KNOWLEDGE**														
Professional category	***100.0% (1,248)***	1.5% (19)	3.8% (48)	2.0% (25)	23.7% (296)	68.9% (860)	1.8% (23)	6.2% (78)	2.0% (25)	34.9% (435)	55.0% (687)	25.2% (315)	65.1% (812)	9.7% (121)
Inactive	100.0% (217)	0.9% (2)	3.2% (7)	4.1% (9)	28.6% (62)	63.1% (137)	3.7% (8)	5.5% (12)	4.1% (9)	35.0% (76)	51.6% (112)	26.3% (57)	63.6% (138)	10.1% (22)
Lower-level occupation	100.0% (542)	1.1% (6)	4.4% (24)	1.3% (7)	24.2% (131)	69.0% (374)	1.1% (6)	6.5% (35)	2.0% (11)	34.7% (188)	55.7% (302)	24.2% (131)	65.7% (356)	10.1% (55)
Higher-level occupation	100.0% (489)	2.2% (11)	3.5% (17)	1.8% (9)	21.1% (103)	71.4% (349)	1.8% (9)	6.3% (31)	1.0% (5)	35.0% (171)	55.8% (273)	26.0% (127)	65.0% (318)	9.0% (44)
**p-value**		**0.055* (0.08)**					**0.089* (0.07)**					0.918		
Highest educational level	***100.0% (1,633)***	1.5% (25)	3.1% (51)	2.1% (34)	21.7% (354)	71.6% (1,169)	2.0% (33)	5.6% (91)	2.7% (44)	34.7% (564)	55.0% (895)	27.2% (442)	64.1% (1,043)	8.7% (141)
Below high school diploma	100.0% (256)	1.6% (4)	3.9% (10)	3.5% (9)	24.2% (62)	66.8% (171)	3.2% (8)	6.3% (16)	5.1% (13)	39.9% (101)	45.5% (115)	30.4% (77)	61.7% (156)	7.9% (20)
High school to 3-year university degree	100.0% (944)	1.7% (16)	3.1% (29)	1.7% (16)	22.2% (210)	71.3% (673)	1.8% (17)	5.6% (53)	2.7% (25)	33.5% (316)	56.4% (532)	26.3% (248)	64.4% (607)	9.2% (87)
4-year university degree or higher	100.0% (433)	1.2% (5)	2.8% (12)	2.1% (9)	18.9% (82)	75.1% (325)	1.9% (8)	5.1% (22)	1.4% (6)	34.1% (147)	57.5% (248)	27.1% (117)	65.0% (280)	7.9% (34)
**p-value**		0.394					**0.021** (0.07)**					0.670		
Works in education or training sector	***100.0% (1,515)***	1.4% (21)	3.1% (47)	1.7% (25)	20.9% (317)	72.9% (1,105)	1.9% (28)	5.5% (83)	2.2% (34)	33.6% (509)	56.8% (859)	26.5% (401)	64.9% (981)	8.5% (129)
No	100.0% (1,426)	1.3% (19)	3.2% (45)	1.5% (22)	21.1% (301)	72.9% (1,039)	1.8% (26)	5.6% (80)	2.2% (32)	33.0% (470)	57.3% (816)	26.2% (373)	65.0% (925)	8.7% (124)
Yes	100.0% (89)	2.2% (2)	2.2% (2)	3.4% (3)	18.0% (16)	74.2% (66)	2.2% (2)	3.4% (3)	2.2% (2)	43.8% (39)	48.3% (43)	31.5% (28)	62.9% (56)	5.6% (5)
**p-value**		0.447[‡]					0.242[‡]					0.393		
Works in scientific sector	***100.0% (1,515)***	1.4% (21)	3.1% (47)	1.7% (25)	20.9% (317)	72.9% (1,105)	1.9% (28)	5.5% (83)	2.2% (34)	33.6% (509)	56.8% (859)	26.5% (401)	64.9% (981)	8.5% (129)
No	100.0% (1,279)	1.5% (20)	3.5% (46)	1.8% (24)	20.7% (270)	72.4% (943)	2.0% (26)	5.7% (73)	2.5% (32)	33.2% (423)	56.6% (722)	25.9% (330)	65.9% (840)	8.2% (105)
Yes	100.0% (236)	0.5% (1)	0.5% (1)	0.5% (1)	22.2% (47)	76.4% (162)	0.8% (2)	4.2% (10)	0.8% (2)	36.3% (86)	57.8% (137)	30.1% (71)	59.7% (141)	10.2% (24)
**p-value**		0.274					0.243					0.187		
Works in healthcare sector	***100.0% (1,515)***	1.4% (21)	3.1% (47)	1.7% (25)	20.9% (317)	72.9% (1,105)	1.9% (28)	5.5% (83)	2.2% (34)	33.6% (509)	56.8% (859)	26.5% (401)	64.9% (981)	8.5% (129)
No	100.0% (1,303)	1.5% (19)	3.4% (44)	1.8% (23)	21.0% (268)	72.3% (925)	2.0% (26)	5.8% (75)	2.5% (33)	32.9% (428)	56.8% (738)	25.6% (333)	66.1% (859)	8.2% (107)
Yes	100.0% (212)	0.8% (2)	1.3% (3)	0.8% (2)	20.8% (49)	76.3% (180)	0.9% (2)	3.8% (8)	0.5% (1)	38.0% (81)	56.8% (121)	32.1% (68)	57.5% (122)	10.4% (22)
**p-value**		**0.047** (0.08)**					0.118					**0.053* (0.06)**		
Previous awareness of NBS	***100.0% (1,632)***	1.5% (25)	3.1% (51)	2.1% (34)	21.8% (356)	71.4% (1,166)	2.0% (33)	5.7% (92)	2.8% (45)	34.5% (561)	55.1% (896)	27.0% (439)	64.3% (1,045)	8.7% (141)
Not knowing NBS	100.0% (792)	1.6% (13)	2.9% (23)	3.5% (28)	23.4% (185)	68.6% (543)	2.7% (21)	5.6% (44)	3.3% (26)	35.3% (279)	53.2% (420)	27.4% (216)	62.8% (495)	9.8% (77)
Knowing NBS	100.0% (840)	1.4% (12)	3.3% (28)	0.7% (6)	20.4% (171)	74.2% (623)	1.4% (12)	5.7% (48)	2.3% (19)	33.7% (282)	56.9% (476)	26.6% (223)	65.7% (550)	7.6% (64)
**p-value**		**<0.001**** (0.11)**					0.208					0.255		
Family history of rare disease	***100.0% (1,510)***	1.5% (23)	3.0% (45)	1.8% (27)	21.8% (329)	71.9% (1,086)	2.1% (31)	5.9% (89)	2.3% (34)	34.1% (515)	55.7% (842)	27.0% (408)	64.4% (972)	8.5% (129)
Concerned	100.0% (215)	3.3% (7)	3.7% (8)	2.3% (5)	20.9% (45)	69.8% (150)	1.4% (3)	7.0% (15)	1.4% (3)	25.6% (55)	64.7% (139)	17.2% (37)	69.3% (149)	13.5% (29)
Not concerned	100.0% (1,295)	1.2% (16)	2.9% (37)	1.7% (22)	21.9% (284)	72.3% (936)	2.2% (28)	5.7% (74)	2.4% (31)	35.5% (460)	54.2% (703)	28.7% (371)	63.6% (823)	7.7% (100)
**p-value**		0.195					**0.030** (0.08)**					**<0.001**** (0.11)**		
**FAMILY AND HOUSEHOLD SITUATION**														
Age of the youngest child	***100.0% (1,638)***	1.5% (25)	3.1% (51)	2.1% (34)	21.7% (356)	71.6% (1,172)	2.0% (33)	5.6% (92)	2.8% (45)	34.6% (565)	55.0% (898)	27.2% (443)	64.2% (1,047)	8.6% (141)
< 1 week	100.0% (402)	1.7% (7)	1.0% (4)	2.2% (9)	15.7% (63)	79.4% (319)	3.0% (12)	3.5% (14)	5.0% (20)	34.0% (135)	54.4% (216)	32.9% (130)	61.8% (244)	5.3% (21)
1 week - < 1 year	100.0% (414)	2.9% (12)	3.6% (15)	1.2% (5)	22.9% (95)	69.3% (287)	1.7% (7)	6.5% (27)	2.7% (11)	37.2% (154)	51.9% (215)	27.1% (112)	63.8% (264)	9.2% (38)
1 - < 2 years	100.0% (423)	1.2% (5)	4.5% (19)	1.7% (7)	24.8% (105)	67.8% (287)	2.4% (10)	7.1% (30)	0.9% (4)	32.6% (138)	57.0% (241)	22.9% (97)	68.3% (289)	8.7% (37)

		**Screening my baby for more diseases at birth seems to me ...**					**Screening my baby for more diseases at birth, using genetic tests (DNA analysis), seems to me …**					**Individual opinion shift with mention of genetic testing**		
		Completely unacceptable	Unacceptable	No opinion	Acceptable	Completely acceptable	Completely unacceptable	Unacceptable	No opinion	Acceptable	Completely acceptable	Negatif shift	No shift	Positive shift
**Characteristic**	**Total**[1]	N = 25	N = 51	N = 34	N = 355	N = 1,166	N = 33	N = 91	N = 44	N = 565	N = 898	N = **443**	**N = 1,047**	**N = 141**
2 - 3 years	100.0% (399)	0.3% (1)	3.3% (13)	3.3% (13)	23.3% (93)	69.9% (279)	1.0% (4)	5.3% (21)	2.5% (10)	34.6% (138)	56.6% (226)	26.1% (104)	62.7% (250)	11.3% (45)
**p-value**		**<0.001**** (0.09)**					**0.014** (0.07)**					**0.007*** (0.07)**		
Number of children	***100.0% (1,571)***	1.5% (23)	3.0% (47)	1.7% (27)	21.6% (340)	72.2% (1,134)	1.9% (30)	5.3% (83)	2.2% (34)	34.8% (545)	55.8% (875)	26.9% (422)	64.5% (1,010)	8.6% (135)
1 child	100.0% (669)	1.8% (12)	3.4% (23)	1.3% (9)	20.5% (137)	72.9% (488)	1.3% (9)	6.7% (45)	2.2% (15)	34.2% (228)	55.5% (370)	28.5% (190)	62.8% (419)	8.7% (58)
2 children	100.0% (561)	1.2% (7)	2.9% (16)	1.6% (9)	21.6% (121)	72.7% (408)	2.5% (14)	3.8% (21)	2.1% (12)	35.6% (199)	56.0% (313)	26.3% (147)	65.5% (366)	8.2% (46)
3 or more children	100.0% (341)	1.2% (4)	2.3% (8)	2.6% (9)	24.0% (82)	69.8% (238)	2.1% (7)	5.0% (17)	2.1% (7)	34.6% (118)	56.3% (192)	24.9% (85)	66.0% (225)	9.1% (31)
**p-value**		0.672					0.471					0.759		
Perceived household budget	***100.0% (1,567)***	1.5% (23)	3.3% (51)	1.7% (26)	21.5% (337)	72.1% (1,130)	1.9% (30)	5.5% (86)	2.0% (31)	34.4% (538)	56.2% (879)	26.4% (412)	64.9% (1,015)	8.7% (136)
Struggling	100.0% (152)	1.3% (2)	3.3% (5)	2.6% (4)	25.0% (38)	67.8% (103)	2.6% (4)	2.6% (4)	0.7% (1)	36.2% (55)	57.9% (88)	19.7% (30)	69.1% (105)	11.2% (17)
Needs monitoring	100.0% (436)	0.9% (4)	4.1% (18)	0.7% (3)	22.9% (100)	71.3% (311)	1.8% (8)	5.1% (22)	2.8% (12)	36.4% (158)	53.9% (234)	27.0% (117)	65.2% (283)	7.8% (34)
Balanced	100.0% (718)	1.0% (7)	3.1% (22)	2.1% (15)	22.6% (162)	71.3% (512)	1.5% (11)	6.3% (45)	1.8% (13)	35.8% (257)	54.6% (392)	28.5% (204)	62.6% (449)	8.9% (64)
Comfortable	100.0% (261)	3.8% (10)	2.3% (6)	1.5% (4)	14.2% (37)	78.2% (204)	2.7% (7)	5.8% (15)	1.9% (5)	26.2% (68)	63.5% (165)	23.5% (61)	68.5% (178)	8.1% (21)
**p-value**		**0.007*** (0.08)** ‡					0.140					0.260		
Household size	***100.0% (1,614)***	1.4% (23)	3.2% (51)	1.8% (29)	21.8% (352)	71.8% (1,159)	2.0% (32)	5.5% (89)	2.5% (40)	34.6% (557)	55.5% (894)	27.1% (436)	64.2% (1,034)	8.7% (140)
1-2 people	100.0% (165)	4.2% (7)	7.3% (12)	1.2% (2)	22.4% (37)	64.8% (107)	3.0% (5)	6.7% (11)	1.8% (3)	38.8% (64)	49.7% (82)	24.2% (40)	64.2% (106)	11.5% (19)
3 or more people	100.0% (1,449)	1.1% (16)	2.7% (39)	1.9% (27)	21.7% (315)	72.6% (1,052)	1.9% (27)	5.4% (78)	2.6% (37)	34.1% (493)	56.1% (812)	27.4% (396)	64.2% (928)	8.4% (121)
**p-value**		**<0.001**** (0.12)**					0.430					0.328		
Marital status	***100.0% (1,615)***	1.4% (23)	3.2% (51)	1.9% (31)	21.7% (351)	71.8% (1,159)	2.0% (33)	5.5% (88)	2.5% (40)	34.6% (558)	55.4% (894)	26.9% (434)	64.4% (1,038)	8.6% (139)
In a couple	100.0% (1,494)	1.3% (20)	2.7% (40)	1.7% (25)	21.6% (323)	72.7% (1,086)	1.9% (28)	5.1% (76)	2.5% (37)	34.4% (513)	56.2% (838)	26.9% (401)	64.7% (964)	8.4% (125)
Single parent	100.0% (121)	2.5% (3)	9.1% (11)	5.0% (6)	23.1% (28)	60.3% (73)	4.1% (5)	9.9% (12)	2.5% (3)	37.2% (45)	46.3% (56)	27.3% (33)	61.2% (74)	11.6% (14)
**p-value**		**<0.001**** (0.12)** ‡					**0.044* (0.08)**					0.464		

	I would like more diseases to be screened at birth for my baby (TFA, Affective Attitude)						Screening more diseases at birth for my baby can improve their health (TFA, Perceived Efficacy)						Screening more diseases at birth for my baby raises moral or ethical concerns (TFA, Ethicality)					
	Strongly disagree	Somewhat disagree	No opinion	Somewhat agree	Strongly agree	**Total**	Strongly disagree	Somewhat disagree	No opinion	Somewhat agree	Strongly agree	**Total**	Strongly disagree	Somewhat disagree	No opinion	Somewhat agree	Strongly agree	**Total**
INDIVIDUAL CHARACTERISTICS																		
**Gender of respondant**	**23 (1.4%)**	**94 (5.8%)**	**66 (4.1%)**	**477 (30%)**	**949 (59%)**	**1,609 (100%)**	**26 (1.6%)**	**63 (3.9%)**	**29 (1.8%)**	**419 (26%)**	**1,072 (67%)**	**1,609 (100%)**	**544 (34%)**	**464 (29%)**	**94 (5.8%)**	**285 (18%)**	**220 (14%)**	**1,607 (100%)**
Female	16 (1.4%)	61 (5.4%)	49 (4.3%)	322 (28%)	682 (60%)	**1,130 (100%)**	14 (1.2%)	42 (3.7%)	22 (1.9%)	285 (25%)	767 (68%)	**1,130 (100%)**	378 (34%)	326 (29%)	72 (6.4%)	190 (17%)	162 (14%)	**1,128 (100%)**
Male	7 (1.5%)	33 (6.9%)	17 (3.5%)	155 (32%)	267 (56%)	**479 (100%)**	12 (2.5%)	21 (4.4%)	7 (1.5%)	134 (28%)	305 (64%)	**479 (100%)**	166 (35%)	138 (29%)	22 (4.6%)	95 (20%)	58 (12%)	**479 (100%)**
p-value	*0.319*						*0.197*						*0.296*					
**Country of birth**	**22 (1.5%)**	**88 (5.8%)**	**61 (4.0%)**	**448 (30%)**	**888 (59%)**	**1,507 (100%)**	**25 (1.7%)**	**55 (3.6%)**	**27 (1.8%)**	**396 (26%)**	**1,004 (67%)**	**1,507 (100%)**	**511 (34%)**	**445 (30%)**	**91 (6.0%)**	**258 (17%)**	**200 (13%)**	**1,505 (100%)**
France	19 (1.4%)	79 (5.7%)	55 (4.0%)	405 (29%)	816 (59%)	**1,374 (100%)**	21 (1.5%)	50 (3.6%)	23 (1.7%)	356 (26%)	924 (67%)	**1,374 (100%)**	460 (34%)	409 (30%)	82 (6.0%)	237 (17%)	184 (13%)	**1,372 (100%)**
Foreign country	3 (2.3%)	9 (6.8%)	6 (4.5%)	43 (32%)	72 (54%)	**133 (100%)**	4 (3.0%)	5 (3.8%)	4 (3.0%)	40 (30%)	80 (60%)	**133 (100%)**	51 (38%)	36 (27%)	9 (6.8%)	21 (16%)	16 (12%)	**133 (100%)**
p-value	*0.768*						*0.249 ‡*						*0.810*					
**Age of respondant**	**23 (1.4%)**	**94 (5.7%)**	**72 (4.4%)**	**489 (30%)**	**958 (59%)**	**1,636 (100%)**	**26 (1.6%)**	**63 (3.9%)**	**34 (2.1%)**	**432 (26%)**	**1,081 (66%)**	**1,636 (100%)**	**544 (33%)**	**474 (29%)**	**103 (6.3%)**	**289 (18%)**	**224 (14%)**	**1,634 (100%)**
< 25 years	2 (2.2%)	7 (7.8%)	4 (4.4%)	29 (32%)	48 (53%)	**90 (100%)**	4 (4.4%)	2 (2.2%)	3 (3.3%)	21 (23%)	60 (67%)	**90 (100%)**	17 (19%)	24 (27%)	11 (12%)	19 (21%)	19 (21%)	**90 (100%)**
25-35 years	11 (1.4%)	50 (6.2%)	32 (4.0%)	253 (31%)	460 (57%)	**806 (100%)**	9 (1.1%)	40 (5.0%)	14 (1.7%)	207 (26%)	536 (67%)	**806 (100%)**	235 (29%)	241 (30%)	53 (6.6%)	159 (20%)	116 (14%)	**804 (100%)**
> 35 years	10 (1.4%)	37 (5.0%)	36 (4.9%)	207 (28%)	450 (61%)	**740 (100%)**	13 (1.8%)	21 (2.8%)	17 (2.3%)	204 (28%)	485 (66%)	**740 (100%)**	292 (39%)	209 (28%)	39 (5.3%)	111 (15%)	89 (12%)	**740 (100%)**
p-value	*0.663*						*0.102*						***<0.001**** (0.10)***					
HEALTH LITERACY AND PRIOR KNOWLEDGE																		
**Professional category**	**20 (1.6%)**	**84 (6.7%)**	**50 (4.0%)**	**374 (30%)**	**720 (58%)**	**1,248 (100%)**	**24 (1.9%)**	**55 (4.4%)**	**23 (1.8%)**	**342 (27%)**	**804 (64%)**	**1,248 (100%)**	**397 (32%)**	**380 (30%)**	**59 (4.7%)**	**233 (19%)**	**179 (14%)**	**1,248 (100%)**
Not working	4 (1.8%)	13 (6.0%)	14 (6.5%)	61 (28%)	125 (58%)	**217 (100%)**	3 (1.4%)	11 (5.1%)	10 (4.6%)	52 (24%)	141 (65%)	**217 (100%)**	47 (22%)	53 (24%)	16 (7.4%)	52 (24%)	49 (23%)	**217 (100%)**
Lower occupational class	6 (1.1%)	35 (6.5%)	19 (3.5%)	180 (33%)	302 (56%)	**542 (100%)**	11 (2.0%)	19 (3.5%)	5 (0.9%)	166 (31%)	341 (63%)	**542 (100%)**	161 (30%)	171 (32%)	30 (5.5%)	101 (19%)	79 (15%)	**542 (100%)**
Upper occupational class	10 (2.0%)	36 (7.4%)	17 (3.5%)	133 (27%)	293 (60%)	**489 (100%)**	10 (2.0%)	25 (5.1%)	8 (1.6%)	124 (25%)	322 (66%)	**489 (100%)**	189 (39%)	156 (32%)	13 (2.7%)	80 (16%)	51 (10%)	**489 (100%)**
p-value	*0.255*						***0.0222** (0.08)***						***<0.001**** (0.14)***					
**Highest education level**	**23 (1.4%)**	**94 (5.8%)**	**73 (4.5%)**	**486 (30%)**	**958 (59%)**	**1,634 (100%)**	**26 (1.6%)**	**63 (3.9%)**	**35 (2.1%)**	**429 (26%)**	**1,081 (66%)**	**1,634 (100%)**	**544 (33%)**	**472 (29%)**	**102 (6.3%)**	**289 (18%)**	**225 (14%)**	**1,632 (100%)**
Less than high school	4 (1.6%)	16 (6.3%)	15 (5.9%)	80 (31%)	141 (55%)	**256 (100%)**	5 (2.0%)	13 (5.1%)	10 (3.9%)	70 (27%)	158 (62%)	**256 (100%)**	51 (20%)	48 (19%)	34 (13%)	65 (25%)	57 (22%)	**255 (100%)**
High school to bachelor's	16 (1.7%)	54 (5.7%)	35 (3.7%)	294 (31%)	546 (58%)	**945 (100%)**	15 (1.6%)	34 (3.6%)	16 (1.7%)	261 (28%)	619 (66%)	**945 (100%)**	303 (32%)	296 (31%)	54 (5.7%)	164 (17%)	127 (13%)	**944 (100%)**
Master's or higher	3 (0.7%)	24 (5.5%)	23 (5.3%)	112 (26%)	271 (63%)	**433 (100%)**	6 (1.4%)	16 (3.7%)	9 (2.1%)	98 (23%)	304 (70%)	**433 (100%)**	190 (44%)	128 (30%)	14 (3.2%)	60 (14%)	41 (9.5%)	**433 (100%)**
p-value	*0.255*						*0.203*						***<0.001**** (0.17)***					
**Working in teaching field**	**22 (1.5%)**	**88 (5.8%)**	**56 (3.7%)**	**450 (30%)**	**901 (59%)**	**1,517 (100%)**	**23 (1.5%)**	**56 (3.7%)**	**22 (1.5%)**	**401 (26%)**	**1,015 (67%)**	**1,517 (100%)**	**529 (35%)**	**447 (30%)**	**85 (5.6%)**	**255 (17%)**	**199 (13%)**	**1,515 (100%)**
No	21 (1.5%)	81 (5.7%)	46 (3.2%)	425 (30%)	855 (60%)	**1,428 (100%)**	22 (1.5%)	53 (3.7%)	20 (1.4%)	374 (26%)	959 (67%)	**1,428 (100%)**	492 (35%)	418 (29%)	80 (5.6%)	246 (17%)	190 (13%)	**1,426 (100%)**
Yes	1 (1.1%)	7 (7.9%)	10 (11%)	25 (28%)	46 (52%)	**89 (100%)**	1 (1.1%)	3 (3.4%)	2 (2.2%)	27 (30%)	56 (63%)	**89 (100%)**	37 (42%)	29 (33%)	5 (5.6%)	9 (10%)	9 (10%)	**89 (100%)**
p-value	***0.00256*** (0.10)***						*0.783 ‡*						*0.320*					
**Working in science**	**22 (1.5%)**	**88 (5.8%)**	**56 (3.7%)**	**450 (30%)**	**901 (59%)**	**1,517 (100%)**	**23 (1.5%)**	**56 (3.7%)**	**22 (1.5%)**	**401 (26%)**	**1,015 (67%)**	**1,517 (100%)**	**529 (35%)**	**447 (30%)**	**85 (5.6%)**	**255 (17%)**	**199 (13%)**	**1,515 (100%)**
No	20 (1.6%)	77 (6.0%)	48 (3.8%)	387 (30%)	748 (58%)	**1,280 (100%)**	21 (1.6%)	45 (3.5%)	20 (1.6%)	343 (27%)	851 (66%)	**1,280 (100%)**	428 (33%)	386 (30%)	72 (5.6%)	217 (17%)	175 (14%)	**1,278 (100%)**
Yes	2 (0.8%)	11 (4.6%)	8 (3.4%)	63 (27%)	153 (65%)	**237 (100%)**	2 (0.8%)	11 (4.6%)	2 (0.8%)	58 (24%)	164 (69%)	**237 (100%)**	101 (43%)	61 (26%)	13 (5.5%)	38 (16%)	24 (10%)	**237 (100%)**
p-value	*0.463*						*0.581*						***0.0855* (0.07)***					
**Working in health**	**22 (1.5%)**	**88 (5.8%)**	**56 (3.7%)**	**450 (30%)**	**901 (59%)**	**1,517 (100%)**	**23 (1.5%)**	**56 (3.7%)**	**22 (1.5%)**	**401 (26%)**	**1,015 (67%)**	**1,517 (100%)**	**529 (35%)**	**447 (30%)**	**85 (5.6%)**	**255 (17%)**	**199 (13%)**	**1,515 (100%)**
No	21 (1.6%)	77 (5.9%)	49 (3.8%)	392 (30%)	765 (59%)	**1,304 (100%)**	22 (1.7%)	46 (3.5%)	21 (1.6%)	346 (27%)	869 (67%)	**1,304 (100%)**	444 (34%)	389 (30%)	73 (5.6%)	218 (17%)	178 (14%)	**1,302 (100%)**
Yes	1 (0.5%)	11 (5.2%)	7 (3.3%)	58 (27%)	136 (64%)	**213 (100%)**	1 (0.5%)	10 (4.7%)	1 (0.5%)	55 (26%)	146 (69%)	**213 (100%)**	85 (40%)	58 (27%)	12 (5.6%)	37 (17%)	21 (9.9%)	**213 (100%)**
p-value	*0.517*						*0.374*						*0.371*					
**Awareness of NBS before survey**	**23 (1.4%)**	**94 (5.8%)**	**73 (4.5%)**	**486 (30%)**	**958 (59%)**	**1,634 (100%)**	**26 (1.6%)**	**63 (3.9%)**	**35 (2.1%)**	**429 (26%)**	**1,081 (66%)**	**1,634 (100%)**	**544 (33%)**	**473 (29%)**	**103 (6.3%)**	**289 (18%)**	**223 (14%)**	**1,632 (100%)**
Not knowing NBS	8 (1.0%)	51 (6.4%)	44 (5.5%)	245 (31%)	446 (56%)	**794 (100%)**	10 (1.3%)	31 (3.9%)	26 (3.3%)	220 (28%)	507 (64%)	**794 (100%)**	222 (28%)	229 (29%)	59 (7.4%)	165 (21%)	119 (15%)	**794 (100%)**
Knowing NBS	15 (1.8%)	43 (5.1%)	29 (3.5%)	241 (29%)	512 (61%)	**840 (100%)**	16 (1.9%)	32 (3.8%)	9 (1.1%)	209 (25%)	574 (68%)	**840 (100%)**	322 (38%)	244 (29%)	44 (5.3%)	124 (15%)	104 (12%)	**838 (100%)**
p-value	***0.0566* (0.07)***						***0.0192** (0.07)***						***<0.001**** (0.13)***					
**Family history of rare disease**	**22 (1.5%)**	**87 (5.8%)**	**63 (4.2%)**	**454 (30%)**	**886 (59%)**	**1,512 (100%)**	**24 (1.6%)**	**58 (3.8%)**	**28 (1.9%)**	**397 (26%)**	**1,005 (66%)**	**1,512 (100%)**	**527 (35%)**	**440 (29%)**	**85 (5.6%)**	**264 (17%)**	**194 (13%)**	**1,510 (100%)**
Concerned	7 (3.3%)	13 (6.0%)	8 (3.7%)	57 (27%)	130 (60%)	**215 (100%)**	7 (3.3%)	9 (4.2%)	4 (1.9%)	54 (25%)	141 (66%)	**215 (100%)**	81 (38%)	55 (26%)	16 (7.4%)	33 (15%)	30 (14%)	**215 (100%)**
Not concerned	15 (1.2%)	74 (5.7%)	55 (4.2%)	397 (31%)	756 (58%)	**1,297 (100%)**	17 (1.3%)	49 (3.8%)	24 (1.9%)	343 (26%)	864 (67%)	**1,297 (100%)**	446 (34%)	385 (30%)	69 (5.3%)	231 (18%)	164 (13%)	**1,295 (100%)**
p-value	*0.140*						*0.327*						*0.406*					
FAMILY AND HOUSEHOLD SITUATION																		
**Age of the youngest child**	**23 (1.4%)**	**94 (5.7%)**	**73 (4.5%)**	**490 (30%)**	**960 (59%)**	**1,640 (100%)**	**26 (1.6%)**	**63 (3.8%)**	**35 (2.1%)**	**433 (26%)**	**1,083 (66%)**	**1,640 (100%)**	**545 (33%)**	**474 (29%)**	**104 (6.3%)**	**290 (18%)**	**225 (14%)**	**1,638 (100%)**
< 1 week	4 (1.0%)	11 (2.7%)	23 (5.7%)	120 (30%)	246 (61%)	**404 (100%)**	3 (0.7%)	8 (2.0%)	13 (3.2%)	95 (24%)	285 (71%)	**404 (100%)**	151 (38%)	95 (24%)	45 (11%)	62 (15%)	49 (12%)	**402 (100%)**
1 week - < 1 year	11 (2.7%)	26 (6.3%)	13 (3.1%)	123 (30%)	241 (58%)	**414 (100%)**	11 (2.7%)	16 (3.9%)	3 (0.7%)	104 (25%)	280 (68%)	**414 (100%)**	128 (31%)	138 (33%)	16 (3.9%)	80 (19%)	52 (13%)	**414 (100%)**
1 - < 2 years	4 (0.9%)	29 (6.9%)	23 (5.4%)	124 (29%)	243 (57%)	**423 (100%)**	8 (1.9%)	17 (4.0%)	10 (2.4%)	119 (28%)	269 (64%)	**423 (100%)**	136 (32%)	132 (31%)	24 (5.7%)	68 (16%)	63 (15%)	**423 (100%)**
2 - 3 years	4 (1.0%)	28 (7.0%)	14 (3.5%)	123 (31%)	230 (58%)	**399 (100%)**	4 (1.0%)	22 (5.5%)	9 (2.3%)	115 (29%)	249 (62%)	**399 (100%)**	130 (33%)	109 (27%)	19 (4.8%)	80 (20%)	61 (15%)	**399 (100%)**
p-value	***0.0612* (0.06)***						***0.0192** (0.07)***						***<0.001**** (0.09)***					
**Number of children**	**21 (1.3%)**	**91 (5.8%)**	**62 (3.9%)**	**463 (29%)**	**934 (59%)**	**1,571 (100%)**	**23 (1.5%)**	**60 (3.8%)**	**27 (1.7%)**	**410 (26%)**	**1,051 (67%)**	**1,571 (100%)**	**532 (34%)**	**460 (29%)**	**93 (5.9%)**	**271 (17%)**	**213 (14%)**	**1,569 (100%)**
1 child	9 (1.3%)	38 (5.7%)	27 (4.0%)	201 (30%)	394 (59%)	**669 (100%)**	11 (1.6%)	26 (3.9%)	11 (1.6%)	185 (28%)	436 (65%)	**669 (100%)**	230 (34%)	204 (30%)	36 (5.4%)	114 (17%)	85 (13%)	**669 (100%)**
2 children	7 (1.2%)	33 (5.9%)	15 (2.7%)	163 (29%)	343 (61%)	**561 (100%)**	8 (1.4%)	19 (3.4%)	7 (1.2%)	135 (24%)	392 (70%)	**561 (100%)**	191 (34%)	167 (30%)	36 (6.4%)	97 (17%)	69 (12%)	**560 (100%)**
3 or more children	5 (1.5%)	20 (5.9%)	20 (5.9%)	99 (29%)	197 (58%)	**341 (100%)**	4 (1.2%)	15 (4.4%)	9 (2.6%)	90 (26%)	223 (65%)	**341 (100%)**	111 (33%)	89 (26%)	21 (6.2%)	60 (18%)	59 (17%)	**340 (100%)**
p-value	*0.625*						*0.643*						*0.540*					
**Perceived budget**	**23 (1.5%)**	**89 (5.7%)**	**60 (3.8%)**	**464 (30%)**	**932 (59%)**	**1,568 (100%)**	**25 (1.6%)**	**60 (3.8%)**	**27 (1.7%)**	**407 (26%)**	**1,049 (67%)**	**1,568 (100%)**	**533 (34%)**	**455 (29%)**	**87 (5.6%)**	**275 (18%)**	**216 (14%)**	**1,566 (100%)**
Struggling	1 (0.7%)	10 (6.6%)	5 (3.3%)	47 (31%)	89 (59%)	**152 (100%)**	1 (0.7%)	7 (4.6%)	2 (1.3%)	43 (28%)	99 (65%)	**152 (100%)**	38 (25%)	45 (30%)	12 (7.9%)	29 (19%)	28 (18%)	**152 (100%)**
Needs monitoring	3 (0.7%)	27 (6.2%)	20 (4.6%)	135 (31%)	251 (58%)	**436 (100%)**	3 (0.7%)	17 (3.9%)	7 (1.6%)	125 (29%)	284 (65%)	**436 (100%)**	134 (31%)	136 (31%)	24 (5.5%)	78 (18%)	64 (15%)	**436 (100%)**
Balanced	13 (1.8%)	41 (5.7%)	26 (3.6%)	217 (30%)	422 (59%)	**719 (100%)**	15 (2.1%)	22 (3.1%)	13 (1.8%)	189 (26%)	480 (67%)	**719 (100%)**	239 (33%)	214 (30%)	38 (5.3%)	130 (18%)	96 (13%)	**717 (100%)**
Comfortable	6 (2.3%)	11 (4.2%)	9 (3.4%)	65 (25%)	170 (65%)	**261 (100%)**	6 (2.3%)	14 (5.4%)	5 (1.9%)	50 (19%)	186 (71%)	**261 (100%)**	122 (47%)	60 (23%)	13 (5.0%)	38 (15%)	28 (11%)	**261 (100%)**
p-value	*0.546*						*0.217*						***0.0032*** (0.08)***					
**Household size**	**22 (1.4%)**	**94 (5.8%)**	**67 (4.1%)**	**482 (30%)**	**951 (59%)**	**1,616 (100%)**	**25 (1.5%)**	**62 (3.8%)**	**29 (1.8%)**	**426 (26%)**	**1,074 (66%)**	**1,616 (100%)**	**541 (34%)**	**468 (29%)**	**96 (5.9%)**	**286 (18%)**	**223 (14%)**	**1,614 (100%)**
1-2 people	7 (4.2%)	17 (10%)	7 (4.2%)	43 (26%)	91 (55%)	**165 (100%)**	7 (4.2%)	9 (5.5%)	3 (1.8%)	47 (28%)	99 (60%)	**165 (100%)**	56 (34%)	48 (29%)	5 (3.0%)	37 (22%)	19 (12%)	**165 (100%)**
3 or more people	15 (1.0%)	77 (5.3%)	60 (4.1%)	439 (30%)	860 (59%)	**1,451 (100%)**	18 (1.2%)	53 (3.7%)	26 (1.8%)	379 (26%)	975 (67%)	**1,451 (100%)**	485 (33%)	420 (29%)	91 (6.3%)	249 (17%)	204 (14%)	**1,449 (100%)**
p-value	***<0.001**** (0.11)***						***0.0229** (0.08)***						*0.228*					
**Couple status**	**23 (1.4%)**	**91 (5.6%)**	**69 (4.3%)**	**483 (30%)**	**951 (59%)**	**1,617 (100%)**	**24 (1.5%)**	**61 (3.8%)**	**31 (1.9%)**	**427 (26%)**	**1,074 (66%)**	**1,617 (100%)**	**541 (33%)**	**468 (29%)**	**98 (6.1%)**	**285 (18%)**	**223 (14%)**	**1,615 (100%)**
In a couple	17 (1.1%)	83 (5.5%)	59 (3.9%)	443 (30%)	894 (60%)	**1,496 (100%)**	19 (1.3%)	52 (3.5%)	24 (1.6%)	396 (26%)	1,005 (67%)	**1,496 (100%)**	511 (34%)	432 (29%)	92 (6.1%)	257 (17%)	204 (14%)	**1,496 (100%)**
Single parent	6 (5.0%)	8 (6.6%)	10 (8.3%)	40 (33%)	57 (47%)	**121 (100%)**	5 (4.1%)	9 (7.4%)	7 (5.8%)	31 (26%)	69 (57%)	**121 (100%)**	30 (25%)	36 (30%)	6 (5.0%)	28 (24%)	19 (16%)	**119 (100%)**
p-value	***<0.001**** (0.11)***						***0.0025*** (0.12) ‡***						*0.208*					

	Choice of technique				Important criteria for choice of technique			
	Technique A	Technique B	Technique C	Total	Number of disease	Uncertainty risk	Both	Total
INDIVIDUAL CHARACTERISTICS								
**Gender of respondant**	314 (20%)	587 (37%)	703 (44%)	1,604 (100%)	256 (16%)	880 (55%)	467 (29%)	1,603 (100%)
Female	231 (21%)	388 (34%)	507 (45%)	1,126 (100%)	167 (15%)	624 (55%)	334 (30%)	1,125 (100%)
Male	83 (17%)	199 (42%)	196 (41%)	478 (100%)	89 (19%)	256 (54%)	133 (28%)	478 (100%)
p-value	***0.0217** (0.07)***				0.164			
**Country of birth**	293 (19%)	549 (37%)	662 (44%)	1,504 (100%)	237 (16%)	837 (56%)	428 (28%)	1,502 (100%)
France	263 (19%)	509 (37%)	600 (44%)	1,372 (100%)	215 (16%)	764 (56%)	391 (29%)	1,370 (100%)
Foreign country	30 (23%)	40 (30%)	62 (47%)	132 (100%)	22 (17%)	73 (55%)	37 (28%)	132 (100%)
p-value	0.0273				0.957			
**Age of respondant**	320 (20%)	595 (37%)	710 (44%)	1,625 (100%)	257 (16%)	891 (55%)	473 (29%)	1,621 (100%)
< 25 years	19 (22%)	39 (44%)	30 (34%)	88 (100%)	11 (13%)	45 (52%)	31 (36%)	87 (100%)
25-35 years	158 (20%)	284 (35%)	359 (45%)	801 (100%)	124 (16%)	445 (56%)	229 (29%)	798 (100%)
> 35 years	143 (19%)	272 (37%)	321 (44%)	736 (100%)	122 (17%)	401 (54%)	213 (29%)	736 (100%)
p-value	0.403				0.651			
HEALTH LITERACY AND PRIOR KNOWLEDGE								
**Professional category**	250 (20%)	475 (38%)	523 (42%)	1,248 (100%)	203 (16%)	696 (56%)	349 (28%)	1,248 (100%)
Not working	64 (29%)	72 (33%)	81 (37%)	217 (100%)	46 (21%)	98 (45%)	73 (34%)	217 (100%)
Lower occupational class	101 (19%)	220 (41%)	221 (41%)	542 (100%)	80 (15%)	304 (56%)	158 (29%)	542 (100%)
Upper occupational class	85 (17%)	183 (37%)	221 (45%)	489 (100%)	77 (16%)	294 (60%)	118 (24%)	489 (100%)
p-value	***0.002*** (0.08)***				***0.004*** (0.08)***			
**Highest education level**	317 (20%)	593 (37%)	712 (44%)	1,622 (100%)	256 (16%)	890 (55%)	472 (29%)	1,618 (100%)
Less than high school	78 (31%)	91 (36%)	81 (32%)	250 (100%)	63 (25%)	103 (42%)	82 (33%)	248 (100%)
High school to bachelor's	165 (18%)	364 (39%)	412 (44%)	941 (100%)	137 (15%)	518 (55%)	284 (30%)	939 (100%)
Master's or higher	74 (17%)	138 (32%)	219 (51%)	431 (100%)	56 (13%)	269 (62%)	106 (25%)	431 (100%)
p-value	***<0.001**** (0.11)***				***<0.001**** (0.10)***			
**Working in teaching field**	295 (20%)	545 (36%)	671 (44%)	1,511 (100%)	245 (16%)	837 (56%)	425 (28%)	1,507 (100%)
No	283 (20%)	514 (36%)	625 (44%)	1,422 (100%)	235 (17%)	781 (55%)	402 (28%)	1,418 (100%)
Yes	12 (13%)	31 (35%)	46 (52%)	89 (100%)	10 (11%)	56 (63%)	23 (26%)	89 (100%)
p-value	0.230				0.275			
**Working in science**	295 (20%)	545 (36%)	671 (44%)	1,511 (100%)	245 (16%)	837 (56%)	425 (28%)	1,507 (100%)
No	250 (20%)	466 (37%)	559 (44%)	1,275 (100%)	208 (16%)	701 (55%)	362 (28%)	1,271 (100%)
Yes	45 (19%)	79 (33%)	112 (47%)	236 (100%)	37 (16%)	136 (58%)	63 (27%)	236 (100%)
p-value	0.566				0.778			
**Working in health**	295 (20%)	545 (36%)	671 (44%)	1,511 (100%)	245 (16%)	837 (56%)	425 (28%)	1,507 (100%)
No	253 (19%)	472 (36%)	574 (44%)	1,299 (100%)	211 (16%)	720 (56%)	364 (28%)	1,295 (100%)
Yes	42 (20%)	73 (34%)	97 (46%)	212 (100%)	34 (16%)	117 (55%)	61 (29%)	212 (100%)
p-value	0.863				0.979			
**Awareness of NBS before survey**	318 (20%)	593 (37%)	711 (44%)	1,622 (100%)	256 (16%)	889 (55%)	473 (29%)	1,618 (100%)
Not knowing NBS	168 (21%)	292 (37%)	327 (42%)	787 (100%)	126 (16%)	410 (52%)	248 (32%)	784 (100%)
Knowing NBS	150 (18%)	301 (36%)	384 (46%)	835 (100%)	130 (16%)	479 (57%)	225 (27%)	834 (100%)
p-value	0.116				***0.0822* (0.06)***			
**Family history of rare disease**	288 (19%)	544 (36%)	675 (45%)	1,507 (100%)	238 (16%)	845 (56%)	422 (28%)	1,505 (100%)
Concerned	49 (23%)	76 (35%)	90 (42%)	215 (100%)	41 (19%)	116 (54%)	58 (27%)	215 (100%)
Not concerned	239 (18%)	468 (36%)	585 (45%)	1,292 (100%)	197 (15%)	729 (57%)	364 (28%)	1,290 (100%)
p-value	0.317				0.368			
FAMILY AND HOUSEHOLD SITUATION								
**Age of the youngest child**	320 (20%)	595 (37%)	713 (44%)	1,628 (100%)	257 (16%)	893 (55%)	474 (29%)	1,624 (100%)
< 1 week	74 (19%)	123 (31%)	195 (50%)	392 (100%)	57 (15%)	203 (52%)	128 (33%)	388 (100%)
1 week - < 1 year	78 (19%)	163 (39%)	173 (42%)	414 (100%)	65 (16%)	227 (55%)	122 (29%)	414 (100%)
1 - < 2 years	89 (21%)	166 (39%)	168 (40%)	423 (100%)	70 (17%)	230 (54%)	123 (29%)	423 (100%)
2 - 3 years	79 (20%)	143 (36%)	177 (44%)	399 (100%)	65 (16%)	233 (58%)	101 (25%)	399 (100%)
p-value	0.104				0.437			
**Number of children**	306 (20%)	567 (36%)	692 (44%)	1,565 (100%)	247 (16%)	871 (56%)	446 (29%)	1,564 (100%)
1 child	114 (17%)	263 (39%)	291 (44%)	668 (100%)	92 (14%)	383 (57%)	192 (29%)	667 (100%)
2 children	107 (19%)	190 (34%)	261 (47%)	558 (100%)	89 (16%)	319 (57%)	149 (27%)	557 (100%)
3 or more children	85 (25%)	114 (34%)	140 (41%)	339 (100%)	66 (19%)	169 (50%)	105 (31%)	340 (100%)
p-value	***0.0162** (0.06)***				***0.0716* (0.05)***			
**Perceived budget**	311 (20%)	570 (36%)	681 (44%)	1,562 (100%)	254 (16%)	861 (55%)	446 (29%)	1,561 (100%)
Struggling	40 (26%)	46 (30%)	66 (43%)	152 (100%)	26 (17%)	76 (50%)	50 (33%)	152 (100%)
Needs monitoring	88 (20%)	168 (39%)	177 (41%)	433 (100%)	69 (16%)	236 (54%)	129 (30%)	434 (100%)

	Choice of technique				Important criteria for choice of technique			
	Technique A	Technique B	Technique C	**Total**	Number of disease	Uncertainty risk	Both	**Total**
INDIVIDUAL CHARACTERISTICS								
Balanced	121 (17%)	277 (39%)	318 (44%)	716 (100%)	103 (14%)	406 (57%)	205 (29%)	714 (100%)
Comfortable	62 (24%)	79 (30%)	120 (46%)	261 (100%)	56 (21%)	143 (55%)	62 (24%)	261 (100%)
p-value	***0.0166** (0.07)***				0.109			
**Household size**	318 (20%)	589 (37%)	702 (44%)	1,609 (100%)	256 (16%)	880 (55%)	469 (29%)	1,605 (100%)
1-2 people	36 (22%)	77 (47%)	51 (31%)	164 (100%)	31 (19%)	76 (46%)	57 (35%)	164 (100%)
3 or more people	282 (20%)	512 (35%)	651 (45%)	1,445 (100%)	225 (16%)	804 (56%)	412 (29%)	1,441 (100%)
p-value	***0.002*** (0.09)***				***0.0702* (0.06)***			
**Couple status**	317 (20%)	589 (37%)	703 (44%)	1,609 (100%)	256 (16%)	882 (55%)	467 (29%)	1,605 (100%)
In a couple	286 (19%)	534 (36%)	668 (45%)	1,488 (100%)	234 (16%)	825 (56%)	426 (29%)	1,485 (100%)
Single parent	31 (26%)	55 (45%)	35 (29%)	121 (100%)	22 (18%)	57 (48%)	41 (34%)	120 (100%)
p-value	***0.003*** (0.09)***				0.232			

Best regression models using explanatory variables recoded as binary (Agree/Important vs. Not agree/Not important) with TFA constraint

Outcome	Formula	AIC	Max_VIF	N
**ACCEPT_bis**	**A_ATTITUDE_bin + A_EFFICACITE_bin + A_ETHICALITE_bin + PAYS2 + HEALTH + PAMR2 + AGE_E_RECODE + HH2**	**257.7075**	**3.000000**	**1,206**
ACCEPT_bis	A_ATTITUDE_bin + A_EFFICACITE_bin + A_ETHICALITE_bin + SEXE2 + PAYS2 + HEALTH + PAMR2 + AGE_E_RECODE + HH2	258.0659	3.000000	1,204
ACCEPT_bis	A_ATTITUDE_bin + A_EFFICACITE_bin + A_ETHICALITE_bin + PAYS2 + HEALTH + PAMR2 + HH2	258.2122	1.264604	1,206
ACCEPT_bis	A_ATTITUDE_bin + A_EFFICACITE_bin + A_ETHICALITE_bin + SEXE2 + PAYS2 + HEALTH + PAMR2 + HH2	258.7887	1.274770	1,204
ACCEPT_bis	A_ATTITUDE_bin + A_EFFICACITE_bin + A_ETHICALITE_bin + SEXE2 + PAYS2 + HEALTH + PAMR2 + AGE_E_RECODE + HH2 + TECH_CRIT	259.6652	3.000000	1,204
ACCEPT_bis	A_ATTITUDE_bin + A_EFFICACITE_bin + A_ETHICALITE_bin + PAYS2 + HEALTH + PAMR2 + AGE_E_RECODE + HH2 + TECH_CRIT	259.7036	3.000000	1,205
ACCEPT_bis	A_ATTITUDE_bin + A_EFFICACITE_bin + A_ETHICALITE_bin + PAYS2 + HEALTH + PAMR2 + HH2 + TECH_CRIT	260.3252	2.000000	1,205
ACCEPT_bis	A_ATTITUDE_bin + A_EFFICACITE_bin + A_ETHICALITE_bin + SEXE2 + PAYS2 + HEALTH + PAMR2 + HH2 + TECH_CRIT	260.5134	2.000000	1,204
ACCEPT_bis	A_ATTITUDE_bin + A_EFFICACITE_bin + A_ETHICALITE_bin + PAYS2 + HEALTH + PAMR2 + AGE_E_RECODE	260.9013	3.000000	1,208
ACCEPT_bis	A_ATTITUDE_bin + A_EFFICACITE_bin + A_ETHICALITE_bin + PAYS2 + AGE_CAT + HEALTH + PAMR2 + HH2	260.9035	2.000000	1,205
**ACCEPT_GENET_bis**	**A_ATTITUDE_bin + A_EFFICACITE_bin + A_ETHICALITE_bin + PAYS2 + AGE_CAT + TECH_CRIT**	**545.7501**	**2.000000**	**1,345**
ACCEPT_GENET_bis	A_ATTITUDE_bin + A_EFFICACITE_bin + A_ETHICALITE_bin + PAYS2 + AGE_CAT	549.1866	2.000000	1,347
ACCEPT_GENET_bis	A_ATTITUDE_bin + A_EFFICACITE_bin + A_ETHICALITE_bin + PAYS2 + TECH_CRIT	549.2765	2.000000	1,346
ACCEPT_GENET_bis	A_ATTITUDE_bin + A_EFFICACITE_bin + A_ETHICALITE_bin + PAYS2	551.2355	1.274817	1,348
ACCEPT_GENET_bis	A_ATTITUDE_bin + A_EFFICACITE_bin + A_ETHICALITE_bin + AGE_CAT + TECH_CRIT	634.6577	2.000000	1,448
ACCEPT_GENET_bis	A_ATTITUDE_bin + A_EFFICACITE_bin + A_ETHICALITE_bin + AGE_CAT	636.7226	2.000000	1,452
ACCEPT_GENET_bis	A_ATTITUDE_bin + A_EFFICACITE_bin + A_ETHICALITE_bin + TECH_CRIT	637.3132	2.000000	1,450
ACCEPT_GENET_bis	A_ATTITUDE_bin + A_EFFICACITE_bin + A_ETHICALITE_bin	638.6726	1.314308	1,454
**ACCEPT_ter**	**A_ATTITUDE_bin + A_EFFICACITE_bin + A_ETHICALITE_bin + PAYS2 + CSP_CAT + BUDGET**	**996.2001**	**3.000000**	**1,018**
ACCEPT_ter	A_ATTITUDE_bin + A_EFFICACITE_bin + A_ETHICALITE_bin + PAYS2 + CSP_CAT + DNN + BUDGET	997.1534	3.000000	1,018
ACCEPT_ter	A_ATTITUDE_bin + A_EFFICACITE_bin + A_ETHICALITE_bin + PAYS2 + CSP_CAT + AGE_E_RECODE + BUDGET	1,001.4352	3.000000	1,018
ACCEPT_ter	A_ATTITUDE_bin + A_EFFICACITE_bin + A_ETHICALITE_bin + PAYS2 + CSP_CAT + DNN + AGE_E_RECODE + BUDGET	1,002.2927	3.000000	1,018
ACCEPT_ter	A_ATTITUDE_bin + A_EFFICACITE_bin + A_ETHICALITE_bin + PAYS2 + CSP_CAT	1,010.5487	2.000000	1,037
ACCEPT_ter	A_ATTITUDE_bin + A_EFFICACITE_bin + A_ETHICALITE_bin + PAYS2 + CSP_CAT + DNN	1,011.7897	2.000000	1,037
ACCEPT_ter	A_ATTITUDE_bin + A_EFFICACITE_bin + A_ETHICALITE_bin + PAYS2 + CSP_CAT + AGE_E_RECODE	1,015.8763	3.000000	1,037
ACCEPT_ter	A_ATTITUDE_bin + A_EFFICACITE_bin + A_ETHICALITE_bin + PAYS2 + CSP_CAT + DNN + AGE_E_RECODE	1,017.0482	3.000000	1,037
ACCEPT_ter	A_ATTITUDE_bin + A_EFFICACITE_bin + A_ETHICALITE_bin + CSP_CAT + BUDGET	1,045.8482	3.000000	1,058
ACCEPT_ter	A_ATTITUDE_bin + A_EFFICACITE_bin + A_ETHICALITE_bin + CSP_CAT + DNN + BUDGET	1,047.0562	3.000000	1,058
**ACCEPT_GENET_ter**	**A_ATTITUDE_bin + A_EFFICACITE_bin + A_ETHICALITE_bin + AGE_CAT + DIPLOME3 + ENSEIGN + PAMR2 + BUDGET + TECH_CRIT**	**1,453.7127**	**3.000000**	**1,164**
ACCEPT_GENET_ter	A_ATTITUDE_bin + A_EFFICACITE_bin + A_ETHICALITE_bin + DIPLOME3 + ENSEIGN + PAMR2 + BUDGET + TECH_CRIT	1,454.9770	3.000000	1,166
ACCEPT_GENET_ter	A_ATTITUDE_bin + A_EFFICACITE_bin + A_ETHICALITE_bin + AGE_CAT + ENSEIGN + PAMR2 + BUDGET + TECH_CRIT	1,455.3471	3.000000	1,166
ACCEPT_GENET_ter	A_ATTITUDE_bin + A_EFFICACITE_bin + A_ETHICALITE_bin + ENSEIGN + PAMR2 + BUDGET + TECH_CRIT	1,456.9392	3.000000	1,168
ACCEPT_GENET_ter	A_ATTITUDE_bin + A_EFFICACITE_bin + A_ETHICALITE_bin + AGE_CAT + DIPLOME3 + ENSEIGN + PAMR2 + BUDGET	1,463.9707	3.000000	1,165
ACCEPT_GENET_ter	A_ATTITUDE_bin + A_EFFICACITE_bin + A_ETHICALITE_bin + AGE_CAT + ENSEIGN + PAMR2 + BUDGET	1,464.9662	3.000000	1,167
ACCEPT_GENET_ter	A_ATTITUDE_bin + A_EFFICACITE_bin + A_ETHICALITE_bin + DIPLOME3 + ENSEIGN + PAMR2 + BUDGET	1,466.2681	3.000000	1,167
ACCEPT_GENET_ter	A_ATTITUDE_bin + A_EFFICACITE_bin + A_ETHICALITE_bin + ENSEIGN + PAMR2 + BUDGET	1,467.4351	3.000000	1,169
ACCEPT_GENET_ter	A_ATTITUDE_bin + A_EFFICACITE_bin + A_ETHICALITE_bin + AGE_CAT + DIPLOME3 + ENSEIGN + PAMR2 + TECH_CRIT	1,478.6725	2.000000	1,183
ACCEPT_GENET_ter	A_ATTITUDE_bin + A_EFFICACITE_bin + A_ETHICALITE_bin + AGE_CAT + ENSEIGN + PAMR2 + TECH_CRIT	1,480.2308	2.000000	1,185

Best regression models using explanatory variables recoded as binary (Agree/Important vs. Not agree/Not important) without TFA constraint

Outcome	Formula	AIC	Max_VIF	N
**ACCEPT_bis**	**PAYS2 + HEALTH + PAMR2 + AGE_E_RECODE + HH2 + A_ATTITUDE_bin + A_EFFICACITE_bin + A_ETHICALITE_bin**	**257.7075**	**3.000000**	**1,206**
ACCEPT_bis	SEXE2 + PAYS2 + HEALTH + PAMR2 + AGE_E_RECODE + HH2 + A_ATTITUDE_bin + A_EFFICACITE_bin + A_ETHICALITE_bin	258.0659	3.000000	1,204
ACCEPT_bis	PAYS2 + HEALTH + PAMR2 + HH2 + A_ATTITUDE_bin + A_EFFICACITE_bin + A_ETHICALITE_bin	258.2122	1.264604	1,206
ACCEPT_bis	SEXE2 + PAYS2 + HEALTH + PAMR2 + HH2 + A_ATTITUDE_bin + A_EFFICACITE_bin + A_ETHICALITE_bin	258.7887	1.274770	1,204
ACCEPT_bis	SEXE2 + PAYS2 + HEALTH + PAMR2 + AGE_E_RECODE + HH2 + A_ATTITUDE_bin + A_EFFICACITE_bin + A_ETHICALITE_bin + TECH_CRIT	259.6652	3.000000	1,204
ACCEPT_bis	PAYS2 + HEALTH + PAMR2 + AGE_E_RECODE + HH2 + A_ATTITUDE_bin + A_EFFICACITE_bin + A_ETHICALITE_bin + TECH_CRIT	259.7036	3.000000	1,205
ACCEPT_bis	PAYS2 + HEALTH + PAMR2 + HH2 + A_ATTITUDE_bin + A_EFFICACITE_bin	259.9800	1.278504	1,259
ACCEPT_bis	PAYS2 + HEALTH + PAMR2 + AGE_E_RECODE + HH2 + A_ATTITUDE_bin + A_EFFICACITE_bin	260.0749	3.000000	1,259
ACCEPT_bis	SEXE2 + PAYS2 + HEALTH + PAMR2 + AGE_E_RECODE + HH2 + A_ATTITUDE_bin + A_EFFICACITE_bin	260.2856	3.000000	1,257
ACCEPT_bis	PAYS2 + HEALTH + PAMR2 + HH2 + A_ATTITUDE_bin + A_EFFICACITE_bin + A_ETHICALITE_bin + TECH_CRIT	260.3252	2.000000	1,205
**ACCEPT_GENET_bis**	**PAYS2 + AGE_CAT + A_ATTITUDE_bin + A_EFFICACITE_bin + A_ETHICALITE_bin + TECH_CRIT**	**545.7501**	**2.000000**	**1,345**
ACCEPT_GENET_bis	PAYS2 + AGE_CAT + A_ATTITUDE_bin + A_EFFICACITE_bin + A_ETHICALITE_bin	549.1866	2.000000	1,347
ACCEPT_GENET_bis	PAYS2 + A_ATTITUDE_bin + A_EFFICACITE_bin + A_ETHICALITE_bin + TECH_CRIT	549.2765	2.000000	1,346
ACCEPT_GENET_bis	PAYS2 + A_ATTITUDE_bin + A_EFFICACITE_bin + A_ETHICALITE_bin	551.2355	1.274817	1,348
ACCEPT_GENET_bis	PAYS2 + AGE_CAT + A_ATTITUDE_bin + A_EFFICACITE_bin + TECH_CRIT	562.9241	2.000000	1,406
ACCEPT_GENET_bis	PAYS2 + A_ATTITUDE_bin + A_EFFICACITE_bin + TECH_CRIT	565.2848	2.000000	1,407
ACCEPT_GENET_bis	PAYS2 + AGE_CAT + A_ATTITUDE_bin + A_ETHICALITE_bin + TECH_CRIT	565.8686	2.000000	1,350
ACCEPT_GENET_bis	PAYS2 + AGE_CAT + A_ATTITUDE_bin + A_EFFICACITE_bin	565.9442	2.000000	1,409
ACCEPT_GENET_bis	PAYS2 + A_ATTITUDE_bin + A_EFFICACITE_bin	566.8567	1.312291	1,410
ACCEPT_GENET_bis	PAYS2 + A_ATTITUDE_bin + A_ETHICALITE_bin + TECH_CRIT	569.4452	2.000000	1,351
**ACCEPT_ter**	**PAYS2 + CSP_CAT + BUDGET + A_ATTITUDE_bin + A_EFFICACITE_bin + A_ETHICALITE_bin**	**996.2001**	**3.000000**	**1,018**
ACCEPT_ter	PAYS2 + CSP_CAT + DNN + BUDGET + A_ATTITUDE_bin + A_EFFICACITE_bin + A_ETHICALITE_bin	997.1534	3.000000	1,018
ACCEPT_ter	PAYS2 + CSP_CAT + AGE_E_RECODE + BUDGET + A_ATTITUDE_bin + A_EFFICACITE_bin + A_ETHICALITE_bin	1,001.4352	3.000000	1,018
ACCEPT_ter	PAYS2 + CSP_CAT + DNN + AGE_E_RECODE + BUDGET + A_ATTITUDE_bin + A_EFFICACITE_bin + A_ETHICALITE_bin	1,002.2927	3.000000	1,018
ACCEPT_ter	PAYS2 + CSP_CAT + BUDGET + A_ATTITUDE_bin + A_ETHICALITE_bin	1,007.4680	3.000000	1,021
ACCEPT_ter	PAYS2 + CSP_CAT + DNN + BUDGET + A_ATTITUDE_bin + A_ETHICALITE_bin	1,008.7759	3.000000	1,021
ACCEPT_ter	PAYS2 + CSP_CAT + A_ATTITUDE_bin + A_EFFICACITE_bin + A_ETHICALITE_bin	1,010.5487	2.000000	1,037
ACCEPT_ter	PAYS2 + CSP_CAT + DNN + A_ATTITUDE_bin + A_EFFICACITE_bin + A_ETHICALITE_bin	1,011.7897	2.000000	1,037
ACCEPT_ter	PAYS2 + CSP_CAT + AGE_E_RECODE + BUDGET + A_ATTITUDE_bin + A_ETHICALITE_bin	1,013.0665	3.000000	1,021
ACCEPT_ter	PAYS2 + CSP_CAT + DNN + AGE_E_RECODE + BUDGET + A_ATTITUDE_bin + A_ETHICALITE_bin	1,014.3171	3.000000	1,021
**ACCEPT_GENET_ter**	**AGE_CAT + DIPLOME3 + ENSEIGN + PAMR2 + BUDGET + A_ATTITUDE_bin + A_EFFICACITE_bin + A_ETHICALITE_bin + TECH_CRIT**	**1,453.7127**	**3.000000**	**1,164**
ACCEPT_GENET_ter	DIPLOME3 + ENSEIGN + PAMR2 + BUDGET + A_ATTITUDE_bin + A_EFFICACITE_bin + A_ETHICALITE_bin + TECH_CRIT	1,454.9770	3.000000	1,166
ACCEPT_GENET_ter	AGE_CAT + ENSEIGN + PAMR2 + BUDGET + A_ATTITUDE_bin + A_EFFICACITE_bin + A_ETHICALITE_bin + TECH_CRIT	1,455.3471	3.000000	1,166
ACCEPT_GENET_ter	ENSEIGN + PAMR2 + BUDGET + A_ATTITUDE_bin + A_EFFICACITE_bin + A_ETHICALITE_bin + TECH_CRIT	1,456.9392	3.000000	1,168
ACCEPT_GENET_ter	AGE_CAT + DIPLOME3 + ENSEIGN + PAMR2 + BUDGET + A_ATTITUDE_bin + A_ETHICALITE_bin + TECH_CRIT	1,461.5791	3.000000	1,167
ACCEPT_GENET_ter	DIPLOME3 + ENSEIGN + PAMR2 + BUDGET + A_ATTITUDE_bin + A_ETHICALITE_bin + TECH_CRIT	1,462.6866	3.000000	1,169
ACCEPT_GENET_ter	AGE_CAT + ENSEIGN + PAMR2 + BUDGET + A_ATTITUDE_bin + A_ETHICALITE_bin + TECH_CRIT	1,463.4214	3.000000	1,169
ACCEPT_GENET_ter	AGE_CAT + DIPLOME3 + ENSEIGN + PAMR2 + BUDGET + A_ATTITUDE_bin + A_EFFICACITE_bin + A_ETHICALITE_bin	1,463.9707	3.000000	1,165
ACCEPT_GENET_ter	ENSEIGN + PAMR2 + BUDGET + A_ATTITUDE_bin + A_ETHICALITE_bin + TECH_CRIT	1,464.8341	3.000000	1,171
ACCEPT_GENET_ter	AGE_CAT + ENSEIGN + PAMR2 + BUDGET + A_ATTITUDE_bin + A_EFFICACITE_bin + A_ETHICALITE_bin	1,464.9662	3.000000	1,167
