# Supplementary material for: Parental acceptability of newborn screening expansion in the genomic era: A nationwide French survey informed by the Theoretical Framework of Acceptability (SeDeN-p3)
Source: PLoS One. 2026 Jun 15;21(6):e0343754. doi: 10.1371/journal.pone.0343754 (PMC13268192; doi:10.1371/journal.pone.0343754)
Supplement: S4 Table — This supplementary table presents free-text comments organised by thematic categories (rows) and by the screening technique criteria judged important by respondents (columns). This layout allows comparison of thematic patterns across technical preference profiles. (PDF) [file pone.0343754.s004.pdf]

Supplementary material S4

	MAXIMAZING NUMBER OF DISEASES	MINIMIZING UNCERTAINTY RISKS	BOTH
MANAGING UNCERTAINTY AND PARENTAL ANXIETY	Unless uncertainty in the results leads to false negatives, which seems unacceptable.	Risk of making parents feel guilty for a result that turns out to be uncertain.	More reassuring.
		We have seen false positives and false negatives with COVID-19 tests. It’s better not to know than to imagine the worst when it’s actually a mistake.	
		The results must be very reliable, otherwise they are too anxiety-inducing.	
		If my child is going to be tested, I prefer real answers rather than worrying about possible risks.	
		Out of fear.	
		Doing a test that may be inaccurate is pointless and anxiety-inducing.	
		It is important to expand screening, but in a way that provides reliable results; otherwise, stress and anxiety will add further problems that could have been avoided and will change parents’ daily lives.	
		It is better to screen for fewer diseases but have more reliable results—we already have enough anxiety with a newborn without adding potentially false worries.	
		Uncertainty is very detrimental.	
		An error must be horrible to deal with.	
		False results can have a very serious impact on the child’s health but also on the parents’ mental health.	
		The risk with technique A is that people will end up no longer believing in the test because it is too often uncertain, and it also detects more diseases, so more tests need to be repeated to check, creating more stress for parents who are already stressed enough after a birth. It is better to screen for fewer diseases but be more sure if the result is positive.	
		The risk of having erroneous results.	
		The stress of an uncertain result.	
		It is better to detect few diseases but have a reliable test than to be faced with a large panel of potential diseases with uncertainty about the results and more worries on top of what parents of a healthy newborn already experience.	
		It is better to be sure than to get scared for no reason.	
		We have children to see them happy and enjoy life with them; if it is to worry and deprive them of their lives on the pretext that they might be ill one day, that is not a life—neither for the child’s development nor for the parents.	
		Fear of a bad result that would make us stressed for nothing, while the arrival of a newborn is a joy. The disease is very serious, so we need certainty.	
		Why create anxiety in parents when there is too large a margin of error? It is better to be sure about certain diseases, knowing that many will not be detected and will appear, or not, much later in the child's life.	
		For me, uncertainty about the result is equivalent to the absence of a test, with the added disadvantage that it generates illegitimate anxiety if that happens.	
EMPHASIS ON RELIABILITY AND CERTAINTY OF RESULTS	The results are more reliable with technique C.	Testing my child is fine, but the results need to be reliable.	It is very important to have the most reliable results possible.
		It is pointless to do tests with no clear outcome.	I prefer to have a definite answer with certain results.
		For me, it is unacceptable to have a test with limited reliability for this type of screening.	
		It is extremely serious to give results that could turn out to be false in the future.	
		Reliability is needed.	
		It has to be reliable.	
		I prefer to confirm a reliable result.	
		I prefer the results to be reliable.	
		There is no point in doing tests if you can’t be sure you’re not sick when the result is negative.	
		The results are more reliable with technique C.	
		It is better to be certain at the time of screening.	
		It is better to start from a reliable technique and then improve coverage for relatives of patients.	
		I am willing to have reliable results.	

BALANCING THE NUMBER OF DISEASES SCREENED WITH TEST RELIABILITY		I prefer fewer tests but more reliable results.	I do not want to expose my child to risks, but I would like to find the best solution if they were sick.
		A balance is needed.	
		I don’t see the point of screening for many diseases if the results are likely to be wrong.	
		I want as many diseases as possible to be screened, but not at the expense of reliability—so a reasonable compromise would be best.	
		I prefer to be sure about the results, even if only a few diseases are tested.	
PREFERENCE FOR COMBINED OR SEQUENTIAL SCREENING APPROACHES	As soon as a means of verification exists for false positives as a second step (e.g., confirmation of a saliva COVID test by PCR), and professionals are trained to handle this “additional check” without causing too much worry, I am in favor of testing as many as possible.	I prefer several analyses with very reliable results rather than just one analysis with a high risk of error.	Ideally, use technique A first, then C to confirm any positives.
	A then C for positives.	In my opinion, it is better to do several tests, perhaps spaced out over time, that screen for few diseases but in a reliable way.	I would be willing to accept the use of two techniques in order to combine results A and B.
	A then C for positives.	If techniques C and B can be used because they are available, it seems completely logical to use them more; but if only technique A is available, then that is the one to use.	We can start with A, then refine with B or C.
	Even if the results are uncertain, further testing later on can clarify the result.		If the number of diseases screened is low but more reliable, I prefer several samples taken on different days to allow for a thorough analysis.
	It seems important to me to offer broad screening, knowing that if a disease is found, it can be followed up with more precise tests to confirm the diagnosis.		
	The uncertain risk of results can be reassessed, but at least a large number of diseases are being screened; at worst, some can be excluded with further review of the results.		
	It is better to detect a disease and pursue further tests than to miss a disease.		
	If additional tests exist for confirmation.		
DEMAND FOR SCIENTIFIC CLARITY AND PRECISE CRITERIA	If the margin of error is specified.	Without numerical proportions, the words have no meaning. We agree that “high” means more than 1 or 50.	My answer to this question should not be taken into account because it was not based on tangible and numerical elements. What exactly is meant by a high, low, or medium risk of uncertain results? How many diseases are tested with each method?
PERCEIVED CLINICAL UTILITY AND EXPECTED BENEFITS	Prevent serious diseases.		Even if screening does not seem completely reliable to me, it still needs to be done so that affected children can be cared for as early as possible.
	Makes it easier to identify the disease at the onset of symptoms.		
