# Supplementary material for: Parental acceptability of newborn screening expansion in the genomic era: A nationwide French survey informed by the Theoretical Framework of Acceptability (SeDeN-p3)
Source: PLoS One. 2026 Jun 15;21(6):e0343754. doi: 10.1371/journal.pone.0343754 (PMC13268192; doi:10.1371/journal.pone.0343754)
Supplement: S5 Table — S5a: Catalogue of verbatims classified by macro-theme and sub-theme, with definitions (n = 81 unique verbatims) Colours match the palette used in Fig 4. Sub-themes are listed in decreasing order of frequency. When a comment addressed several sub-themes, it was repeated and counted in each relevant category. S5b: Cross-tabulation of general and genomic acceptability among respondents who provided a free-text comment (n = 81 unique verbatims) Rows represent acceptability with genetic testing; columns represent acceptability in general. Each cell shows the count followed by the percentage of the comment subgroup. S5c: Full interpretation of qualitative material and mapping of parental profiles. (PDF) [file pone.0343754.s005.pdf]

Supplementary material S5

S5a

Macro/subtheme	Definition	Verbatims
Perceived benefits (n=34 verbatims)		
Projected clinical benefit (n=13)	Screening is justified by a concrete health gain: earlier treatment, reduced severity, fewer complications	- *I didn't understand before; these diseases are severe but maybe early screening can cure some.*<br>- *If we can prevent each illness earlier, it might be a victory for the child's life.*<br>- *It's necessary to have preventive information.*<br>- *I'd like to know as early as possible if my child is healthy so I can help and prevent disease*<br>- *Knowing whether parents carry a gene lets us treat early or prepare to ease the child's life*<br>- *Prevent serious diseases.*<br>- *Prevention and information are fundamental*<br>- *Preventive screening is needed to avoid all kinds of complications.*<br>- *Seems important, especially if treatments or preventive measures exist — allows earlier monitoring and care.*<br>- *So the child can live long, happy and be supported with proper care*<br>- *The earlier diseases are discovered, the more effective treatments are.*<br>- *We must detect as many diseases or allergies as possible to treat quickly and support overwhelmed parents.*<br>- *You need to know in order to prevent*
Informed decision-making (n=8)	Results are valued as actionable information that empowers parents to choose, act, or adapt care pathways	- *If it lets me choose and doesn't affect the baby, it's important.*<br>- *It's good to be prepared from the start, to organise or fully enjoy life*<br>- *I'd like to know as early as possible if my child is healthy so I can help and prevent disease*<br>- *Knowing whether parents carry a gene lets us treat early or prepare to ease the child's life*<br>- *Plan ahead to help and support.*<br>- *To act with full knowledge*<br>- *To manage the situation better*<br>- *Today's tools let us know earlier whether we're affected; even without treatment earlier awareness helps plan and maybe delay disease*
Valuing knowledge (n=7)	A broad appreciation of 'knowing' for its own sake: reassurance, awareness, and peace of mind	- *Better to know everything.*<br>- *It's better to know.*<br>- *It's important to know.*<br>- *It's reassuring and makes you aware of diseases.*<br>- *I'd like more tests at birth so I know my baby's status*<br>- *Lets us know the risks and predispositions to various diseases.*<br>- *Prevention and information are fundamental*
Early anticipation (n=6)	Emphasis on gaining time: knowing earlier allows parents to plan, organise or simply enjoy life with less uncertainty	- *Having a child is stressful; if we can see future risks we can prepare.*<br>- *I think it's important to run these tests because the earlier the better.*<br>- *It's good to be prepared from the start, to organise or fully enjoy life.*<br>- *Preventing serious future diseases is important because it allows us to prepare and react better when the time comes.*<br>- *Prevention is better than cure.*<br>- *Today's tools let us know earlier whether we're affected; even without treatment earlier awareness helps plan and maybe delay disease.*
Advancing research or valuing innovation (n=3)	The screening is welcomed primarily because it fuels scientific progress and medical innovation	- *Many tests and trials are needed to achieve success*<br>- *Medical progress can only be beneficial*<br>- *The best option to build cohorts and advance research*

- Safety-driven conditions (n=22)		
Non-invasive methods (n=9)	Blood spots or cord blood are fine, but any extra puncture or painful sampling is rejected; "no added suffering" for the newborn is a prerequisite	- *As long as the baby doesn't suffer more than now, that's fine*<br>- *As long as the test is non-invasive*<br>- *Depends on how the DNA is collected*<br>- *If it's not too painful for my child, I have no objection.*<br>- *If the test isn't more invasive I'm in favour each time.*<br>- *Important and easy from the umbilical cord, I think*<br>- *It's easy to do*<br>- *No extra sampling means less suffering for the newborn; parents will be reassured*<br>- *Provided it's non-intrusive for the baby (no puncture).*
Restricted sampling and use (n=6)	Collect only what is strictly needed, use it only for the stated screening purpose, and prohibit any further profiling or research without renewed consent	- *Always afraid of what could be done later with these samples.*<br>- *I don't want every newborn's DNA to be collected.*<br>- *If we're sure it won't be used for anything else*<br>- *I'm not paranoid, but will it be just a genetic test or blood profiling as well?*<br>- *No guarantee the samples are destroyed after testing*<br>- *Provided the sample is used only for screening and never goes into a genetic database.*
Data confidentiality (n=3)	Acceptable only if every sample/result is anonymised, stored temporarily, and destroyed after use so the child's DNA cannot be traced back to them	- *As long as everything is done properly and nothing is kept.*<br>- *Make sure my child's DNA isn't stored in a nominative database after the test*<br>- *Yes, as long as it stays private and is helpful*
Legal safeguards and transparency (n=3)	Respondents want a well-defined legal framework that openly specifies who can access data, for how long, and how it will be destroyed	- *As long as everything is done properly and nothing is kept.*<br>- *Only if it's strictly regulated*<br>- *Testing must be strictly regulated and data retention explained to parents*
Explicit parental consent (n=2)	Screening must be performed solely with parents' clear, informed authorisation, never automatically	- *Always with the parents' consent.*<br>- *With the parents' consent, of course.*
- Evidence or obvious necessity (n=15)		
Evidence or obvious necessity (n=15)	Respondents in the "Evidence / obvious necessity" category framed expanded genomic screening as self-evident - ranging from emphatic claims that it is indispensable or should be mandatory, through straightforward "common-sense" approval, to the simple view that if the test is available one might as well use it.	- *Essential*<br>- *Good*<br>- *Health comes first*<br>- *I completely agree*<br>- *I think it's normal*<br>- *If we have the chance to know what illnesses our baby might face, why not test?*<br>- *It even seems indispensable*<br>- *It's extremely important*<br>- *It's more than important - there should be a genetic test at every birth if possible*<br>- *Necessary*<br>- *Normal*<br>- *These preventive tests seem indispensable to me*<br>- *Useful*<br>- *Very good*<br>- *Well yes, it's better for me*
- Moral or ethical objections (n=9)		
Risk of eugenics (n=3)	Fear that large-scale genomic screening could be used to select, rank or discriminate among children, echoing past eugenic practices.	- *An open door to eugenics*<br>- *Eugenics*<br>- *Feels like a doorway to eugenics*
General ethical vigilance (n=2)	Broad call for caution and strict oversight to prevent misuse or ethical drift as technology and data practices evolve.	- *Afraid of ethical problems*<br>- *Beware of ethical issues and possible drift*

Going too far or moral excess (n=2)	Sense that the programme exceeds reasonable limits of medical intervention; common-sense boundaries should rein in how much we test.	- *This is getting a bit extreme*<br>- *We're going too far; common sense should prevail*
Sanctity of life, "playing God" (n=2)	Moral unease at intervening too deeply in human fate; deciding a newborn's future on the basis of a genetic test is viewed as overstepping the natural order.	- *I find it immoral and inhuman to decide a newborn's life based solely on a test.*<br>- *Stay strictly regulated - we're not God, and dying of a heart attack at 40 is part of life even if it's early.*
- Anticipated psychological impact (n=4)		
Anticipated psychological impact (n=4)	Respondents worry that expansive genomic screening could burden families with prolonged anxiety and vigilance: knowing too early, especially for late-onset conditions or uncertain risks, may overshadow the joy of a newborn's first days and make some parents wish they had remained unaware.	- *Case-by-case testing has value if there's family history, but it may expose families to years of stress and hyper-vigilance before any symptoms. That's no life.*<br>- *It depends on when the disease would manifest. If it's only in adulthood we might as well test later. If parents are not carriers there's little point. Testing can also worry parents—sometimes not knowing until age 15 is better.*<br>- *It's complicated. Medically we want healthy kids, but morally we don't want their first days filled with tests. Those are precious moments. Not every screen is useful; if parents were tested beforehand we could adapt what needs testing.*<br>- *The answers aren't obvious - I'm not sure I'd feel the same if I actually had to test my own children for all these diseases.*

S5b

**Acceptability in general<br><br>with genetics**	**Completely unacceptable**	**Somewhat unacceptable**	**No opinion**	**Somewhat acceptable**	**Completely acceptable**	***Row total***
**Completely unacceptable**	2 (2.5 %) ; Zone 5	0 (0.0 %)	0 (0.0 %)	0 (0.0 %)	1 (1.2 %) ; Zone 4	***3 (3.7 %)***
**Somewhat unacceptable**	0 (0.0 %)	2 (2.5 %) ; Zone 5	0 (0.0 %)	5 (6.2 %) ; Zone 4	2 (2.5 %) ; Zone 4	***9 (11.1 %)***
**No opinion**	0 (0.0 %)	0 (0.0 %)	0 (0.0 %)	0 (0.0 %)	0 (0.0 %)	***0 (0.0 %)***
**Somewhat acceptable**	0 (0.0 %)	0 (0.0 %)	0 (0.0 %)	4 (5.0 %) ; Zone 3	14 (17.3 %) ; Zone 3	***18 (22.2 %)***
**Completely acceptable**	0 (0.0 %)	2 (2.5 %) ; Zone 2	0 (0.0 %)	1 (1.2 %) ; Zone 2	48 (59.3 %) ; Zone 1	***51 (63.0 %)***
Column total	***2 (2.5 %)***	***4 (5.0 %)***	***0 (0.0 %)***	***10 (12.3 %)***	***65 (80.2 %)***	***81 (100 %)***

<u>S5c</u>

A total of 81 respondents (4.9%) provided open-text comments (S5a Table). These were positioned on a bubble matrix according to each respondent's combined eNBS and gNBS ratings (Fig 5). Cross-tabulation is presented in S5b Table. Five qualitative profiles emerged from this mapping. The largest group (Zone 1, n=48) consisted of parents who rated both scenarios as highly acceptable. Their comments were benefit-centred, highlighting earlier treatment, informed decision-making, valuing knowledge and anticipatory planning, often framed as an "obvious necessity," with occasional references to the importance of non-invasive procedures. A smaller set of respondents (Zone 2, n=3) expressed an even stronger endorsement when genetics was specified; for them, the prospect of genomic analysis heightened rather than altered an already favourable view. Another sizeable group (Zone 3, n=18) also maintained positive ratings across both scenarios, but their support was explicitly conditional. Their narratives emphasized requirements such as non-invasiveness, restricted use of samples, strong data confidentiality and clear legal safeguards, often invoking broader principles of proportionality and ethical oversight. By contrast, respondents in Zone 4 (n=8) were favourable to eNBS in general but shifted downward when the genetic dimension was introduced. Their comments pointed to concerns about privacy, fears of eugenics and a perception that existing safeguards were insufficient. Finally, a smaller set of respondents (Zone 5, n=4) consistently rejected both scenarios, invoking ethical or existential arguments such as the sanctity of life, the rejection of medical over-reach and the fear of eugenics. Mentions of anticipated psychological burden (n=4, highlighted in orange in Fig 5) were distributed across profiles and did not cluster within a specific acceptability pattern.
